# A novel scan statistics approach for clustering identification and comparison in binary genomic data

**DOI:** 10.1186/s12859-016-1173-8

**Published:** 2016-09-22

**Authors:** Danilo Pellin, Clelia Di Serio

**Affiliations:** 1 University Center of Statistics for the Biomedical Sciences, Vita-Salute San Raffaele University, Via Olgettina 58, Milan, 20132 Italy; 2 Johann Bernoulli Institute, University of Groningen, Nijenborgh 9, Groningen, 9747 AG Netherlands

**Keywords:** Scan statistics, Viral integration sites, Cluster identification, Binary genomic data

## Abstract

**Background:**

In biomedical research a relevant issue is to identify time intervals or portions of a n-dimensional support where a particular event of interest is more likely to occur than expected. Algorithms that require to specify a-priori number/dimension/length of clusters assumed for the data suffer from a high degree of arbitrariness whenever no precise information are available, and this may strongly affect final estimation on parameters. Within this framework, spatial scan-statistics have been proposed in the literature, representing a valid non-parametric alternative.

**Results:**

We adapt the so called Bernoulli-model scan statistic to the genomic field and we propose a multivariate extension, named Relative Scan Statistics, for the comparison of two series of Bernoulli r.v. defined over a common support, with the final goal of highlighting unshared event rate variations. Using a probabilistic approach based on success probability estimates and comparison (likelihood based), we can exploit an hypothesis testing procedure to identify clusters and relative clusters. Both the univariate and the novel multivariate extension of the scan statistic confirm previously published findings.

**Conclusion:**

The method described in the paper represents a challenging application of scan statistics framework to problem related to genomic data. From a biological perspective, these tools offer the possibility to clinicians and researcher to improve their knowledge on viral vectors integrations process, allowing to focus their attention to restricted over-targeted portion of the genome.

**Electronic supplementary material:**

The online version of this article (doi:10.1186/s12859-016-1173-8) contains supplementary material, which is available to authorized users.

## Background

In many different research areas it is of interest to identify time intervals or portions of a *n*-dimensional support where a particular event is more likely to occur than expected. These regions, which in biology are commonly called *clusters* or *hotspots*, are presumable characterized by an increased probability of success and their identification may throw light on a better understanding of the underlying events-generating process. Different perspectives can be adopted according to both classical and Bayesian frameworks, and within parametric and non-parametric approaches. Applications include also the fields of epidemiology, public health, astronomy and neuroscience, ranging from one to *n*-dimensional spaces [1–6].

Many algorithms require to specify a-priori the number of clusters assumed for the data and/or their expected dimension and/or length. These settings may strongly affect the final estimation results and requires a high degree of arbitrariness on the parameters whenever no precise informations are available. Spatial scan has been proposed with wide success in the literature [5] becoming one of the main epidemiological statistics tools in disease surveillance to test the null hypothesis that geographical data are randomly distributed against a localized cluster alternative. This method and its natural extensions are of particular interest since no prior information on parameters or clusters characteristics are required. Indeed, the scan statistic is able to address any of the following interrelated purposes: a) to test if event aggregation occurs (overall clustering), b) cluster localization (detection of cluster) c) to test event distribution on a specific region (focused test).

In a multivariate setting, a challenging goal for researchers may be the identification of regions where two spatial processes - defined over a common support - show different behaviours. More in detail, the processes are allowed to share fluctuations in probability (or rate) of success. To address this type of problem, a few alternatives have been currently proposed. Most of them rely on non-parametric estimation of relative risk function by means of kernel method, as proposed in [7, 8] for environmental epidemiology data analysis.

Scan statistics methodologies have been proposed for the analysis of Poisson and Gaussian distributed random variables, categorical and many other type data. In this paper we are interested in modeling spatial distribution of a particular type of genomic data, such as viral IS retrieved by using Next Generation Sequencing (NGS) platforms [9, 10]. From a statistical point of view, the genome is interpreted as a set of 2×3×10^9^ independent Bernoulli random variables *B*_*chr,postion,strand*_, where 1 means that a viral integration has been observed mapping to that particular genomic coordinates and 0 otherwise.

In genomics a few alternatives have been proposed to identify clusters of ISs, termed Common Integration Sites (CIS) or hotspots. The most popular in the biological literature is a gene integration frequency based method, involving Grubbs test [11] for outlier identification [12]. This approach suffers from an important limitation since ISs located outside genes and their neighborhoods are excluded from the analysis, thus leading to miss possible important intergenic CISs potentially very informative. To overcome this problem, an alternative method based on DBSCAN [13] algorithm has been proposed in [10]. The main drawback of this algorithm is the strong dependence of results on tuning parameters settings, difficult to calibrate for different sized data sets involving viral vectors with different clustering behaviours. To solve this issue, in [10] authors proposed a framework based on re-sampling in-silico generated ISs to select an optimal distance parameter, by controlling the probability of smaller clusters (3 events) identification. However, the impact of this procedure on bigger clusters investigation is unclear.

*Insertional mutagenesis* [14] provide a good setting in clinical genomics to understand the importance of comparing two integration patterns. This phenomenon is caused by virus integration trajectory within particular dangerous genomic regions, such as oncogenic regions. Since many studies revealed different patterns in site selection process among available viral vectors, a statistical procedure that allows to identify differently targeted regions represents a fundamental tool in limiting insertional mutagenesis risk. Another framework where tools for detecting genomic clustering might be extremely helpful for biological research is the investigation of active regulatory element involved in differentiation process. This can be performed by exploiting the capability of particular viral vectors, such as the *Murine Leukemia Virus* (MLV) derived vectors, in marking transcription start site of active genes [15, 16].

Some approaches have been proposed in the literature [17] based on kernel methods where two separate non-parametric kernel densities are estimated by means of Gaussian kernels. Comparative clusters of integrations (hotspots) can be selected in those genomic areas where no overlapping among confidence intervals for densities were detected. However, the arbitrary choice of smoothing parameters (bandwidth) strongly affects the detecting procedure.

In this paper we propose to overcome several problematic issues in the existing procedures, by extending the Bernoulli model proposed in [5] to the genomic field. We first study more in depth the preliminary results presented in [18] for clusters identification in univariate setting. We also propose a novel multivariate alternative, that we call Relative Scan Statistics for comparing two integration patterns by the identification of *comparative* or *relative clusters*. Multivariate extensions of scan statistics have already been proposed in the literature [19],to detect disease outbreaks by means of simultaneous analysis of different data sets. To our knowledge, there are no paper focusing on detecting *differences* among data sets using scan statistics. Finally, the proposed methods are compared to the existing ones, like the DBSCAN algorithm and the comparative hotspot [17] procedure.

The paper is organized as it follows. In Section Methods we introduce the Kulldorff scan statistics for Bernoulli data, we illustrate how the method can be used to compare two genomic data sets and the algorithm implementation is presented. In Section Results and discussion real data sets are descibed and results obtained for the univariate and multivariate analysis are discussed. Final consideration and conclusion are provided in Section Conclusions.

## Methods

### Kulldorff spatial scan statistics for Bernoulli model

The method proposed by [5] can be adopted to face clusters identification as a general problem. In this work, we focus on Bernoulli model, since we consider a particular type of genomic data – derived by viral vector integration in gene therapy – that reveal presence or absence of a genomic event (namely the integration). A brief description of the underlying idea and the specification of the method for the univariate data analysis previously proposed in [18], is next introduced. Let define the whole study area under investigation as *G*, $\mathcal {Z}$ the collection of zones *Z*⊂*G* obtained by scanning the support by means of a window of variable size.

The spatial scan statistics, *S*, is defined as the maximum likelihood ratio over all possible zone $Z \in \mathcal {Z}$:

1 $$  S= \frac{\max\{ L(Z) \}}{L_{0}} = \max_{Z} \left\lbrace \frac{L(Z)}{L_{0}} \right\rbrace.  $$

*S* simultaneously localizes the $Z \in \mathcal {Z}$ (chromosome, start and end coordinates) providing the maximum evidence for the presence of an hotspot and gives a measure of its goodness of fit with respect to a constant rate null hypothesis. From a computational perspective, to proceed with the calculation of Eq. 1, we need to define the total amount of success and trials available on *G*, respectively *X* and *N*. In addition, conditioning on a specific zone *Z*, *n*_*Z*_ and *x*_*Z*_ are the count of trials and success observed within *Z*. Finally, to identify *S* is necessary to maximize the likelihood:

$$  L\left(Z,p_{Z},q_{Z}\right)\!\propto\! p_{Z}^{x_{Z}} \!(1\!-p_{Z})^{n_{Z}-x_{Z}}\!q_{Z}^{X-x_{Z}}\! (1-q_{Z})^{(N-n_{Z})-(X-x_{Z})}. $$

for all $Z \in \mathcal {Z}$ by means of the following functions:

$$\begin{aligned} L(Z) &= L\left(p_{Z},q_{Z}|Z=Z_{j}\right)= {\left(\frac{x_{Z}}{n_{Z}} \right) }^{x_{Z}} ~ {\left(1- \frac{x_{Z}}{n_{Z}} \right)}^{n_{Z}-x_{Z}}\\ & \quad\times{\left(\frac{X-x_{Z}}{N-n_{Z}} \right)}^{X-x_{Z}} ~ {\left(1- \frac{X-x_{Z}}{N-n_{Z}} \right) }^{(N-n_{Z})-(X-x_{Z})} \end{aligned}  $$

if $\frac {x_{Z}}{n_{Z}} > \frac {X-x_{Z}}{N-n_{Z}} $, and

$$ L(Z) = {\left(\frac{X}{N}\right)}^{X} ~ {\left(\frac{N-X}{N} \right)}^{N-X}\;. $$

otherwise. Under the null hypothesis, corresponding to a constant probability of success over *G*, the likelihood is given by:

$$ L_{0} = {\left(\frac{X}{N} \right)}^{X} ~ {\left(\frac{N-X}{N} \right)}^{N-X}\; $$

for all $Z \in \mathcal {Z}$.

### Multivariate extension to novel relative scan statistics for Bernoulli model

Let now introduce a novel multivariate extension of the described method for identifying the most highly significant *relative cluster*. The method is described as referred to a bivariate case, in order to ensure clarity of the underlying idea, but can be easily extended for the comparison of more than two processes.

We define a *relative cluster* as an area $Z \in \mathcal {Z}$ where two Bernoulli processes show different behaviour, in terms of success probability variation with respect to *Z*^*C*^=*G*∖*Z*. Conditioning on a particular area $Z \in \mathcal {Z}$ let define *p*_*Z*1_ and *p*_*Z*2_ as the probability of being an event within *Z* respectively for *Process*_1_ and *Process*_2_ and *q*_*Z*1_ and *q*_*Z*2_ be referred to *Z*^*C*^. Bernoulli trials location, assumed as known over *G*, can differ between the two processes. All the analyses are conditioned on the total count of observed events *X*_1_ and *X*_2_. The aim is here to highlight regions where the difference between probability of success in the two series is maximum and statistically significant, accounting for possible different data sets size and non-constant but shared underlying probability variations.

To measure and compare within each process the behaviour observed within/outside *Z*, we propose the success probability ratio $\frac {p_{Zi}}{q_{Zi}}$. The ratio takes values in *R*^+^ and more specifically $ 0 \leq \frac {p_{Zi}}{q_{Zi}} < 1$ if the probability of success is lower within *Z* than outside and $1 < \frac {p_{Zi}}{q_{Zi}} < \infty $ otherwise.

Let now define as relative cluster for *Process*_*i*_ with respect to *Process*_*j*_ the region $Z \in \mathcal {Z}$ where the probability ratio $\frac {p_{Zi}}{q_{Zi}}$ is greater than corresponding ratio $\frac {p_{Zj}}{q_{Zj}}$. Conditioning on $Z \in \mathcal {Z}$ it is possible to define hypothesis system as:

$$ \left\{\begin{array}{l} H_{0Z}:\frac{{p_{Z1}}}{{q_{Z1}}}=\frac{{p_{Z2}}}{{q_{Z2}}}\\ H_{1Z}:\frac{{p_{Z1}}}{{q_{Z1}}} \neq \frac{{p_{Z2}}}{{q_{Z2}}} \end{array}\right. $$

or alternatively as:

2 $$  \left\{\begin{array}{l} H_{0Z}:\left\lbrace {p_{Z1}}=k_{Z} {q_{Z1}} \right\rbrace \cap \left\lbrace {p_{Z2}}=k_{Z} {q_{Z2}}\right\rbrace\\ H_{1Z} : \left\lbrace {p_{Z1}} \neq k_{Z} {q_{Z1}} \right\rbrace \cup \left\lbrace {p_{Z2}} \neq k_{Z} {q_{Z2}}\right\rbrace \end{array}\right.  $$

Under the null hypothesis, the probability of success may vary over *G* but it must be shared among processes and characterized by the same value of *k*_*Z*_. To estimate the scan statistics *S*, we first need to define the likelihood ratio conditioned on *Z*. Let now:

- *N*_1_ and *N*_2_ be the total count of Bernoulli trials for each process.
- *X*_1_ and *X*_2_ be the total count of success
- *n*_1*Z*_ and *n*_2*Z*_ be the size, in terms of trials, of the Z with respect of each series
- *x*_1*Z*_ and *x*_2*Z*_ be the success amount within Z with respect of each series

According to biological motivations related to virus integration mechanisms, supported and derived from several studies on IS data analysis, it is reasonable to assume that within each treated cell’s genome, only one integration event can occur [20]. In addition, there are no biologically meaningful reasons to suppose that any interaction between IS events occurs in distinct cells. From a modelling perspective, this is equivalent to assume independence among observations. Even more so, the two series can be assumed to be independent and the likelihood function associated to the joint model corresponds to the product of the likelihoods of each process.

$$ \begin{aligned} &L\left(Z,{p_{Z1}},{q_{Z1}},{p_{Z2}},{q_{Z2}}\right) \propto {{p_{Z1}}}^{x_{1Z}} \left(1-{p_{Z1}}\right)^{n_{1Z}-x_{1Z}}\\ &\qquad{q_{Z1}}^{X_{1}-x_{1Z}} \left(1-{q_{Z1}}\right)^{\left(N_{1}-n_{1Z}\right)-\left(X_{1}-x_{1Z}\right)}\\ &\,\,\,\,\times {p_{Z2}}^{x_{2Z}} \left(1-{p_{Z2}}\right)^{n_{2Z}-x_{2Z}} ~ {q_{Z2}}^{X_{2}-x_{2Z}}\\ &\qquad\left(1-{q_{Z2}}\right)^{\left(N_{2}-n_{2Z}\right)-\left(X_{2}-x_{2Z}\right)} \end{aligned} $$

Conditioned on *Z* = *Z*_*j*_:

3 $$  \begin{aligned} L_{Z_{j}}&=\sup_{H_{1Z}} L({p_{Z1}},{q_{Z1}},{p_{Z2}},{q_{Z2}}|Z = Z_{j})\\ &={\left(\frac{x_{1Z}}{n_{1Z}} \right) }^{x_{1Z}} ~ {\left(1- \frac{x_{1Z}}{n_{1Z}} \right) }^{n_{1Z}-x_{1Z}} {\left(\frac{X_{1}-x_{1Z}}{N_{1}-n_{1Z}} \right) }^{X_{1}-x_{1Z}}\\&\quad\quad{\left(1- \frac{X_{1}-x_{1Z}}{N_{1}-n_{1Z}} \right) }^{(N_{1}-n_{1Z})-(X_{1}-x_{1Z})}\\ &\quad\times {\left(\frac{x_{2Z}}{n_{2Z}} \right) }^{x_{2Z}} ~ {\left(1- \frac{x_{2Z}}{n_{2Z}} \right) }^{n_{2Z}-x_{2Z}} {\left(\frac{X_{2}-x_{2Z}}{N_{2}-n_{2Z}} \right) }^{X_{2}-x_{2Z}}\\&\quad\quad{\left(1- \frac{X_{2}-x_{2Z}}{N_{2}-n_{2Z}} \right) }^{(N_{2}-n_{2Z})-(X_{2}-x_{2Z})} \end{aligned}  $$

The maximum likelihood estimators are given by:

$$ \begin{aligned} \hat{{p_{Z1}}}&=\frac{x_{1Z}}{n_{Z}};~ \hat{{p_{Z2}}}=\frac{x_{2Z}}{n_{Z}}; ~ \hat{{q_{Z1}}}=\frac{X_{1}-x_{1Z}}{N-n_{Z}};\\ \hat{{q_{Z2}}}&=\frac{X_{2}-x_{2Z}}{N-n_{Z}} \;. \end{aligned} $$

By introducing the constraint *p*_*Z*1_=*k*_*Z*_*q*_*Z*1_∩*p*_*Z*2_=*k*_*Z*_*q*_*Z*2_ as defined in the null hypothesis *H*_0*Z*_ in Eq. 2, the likelihood function becomes:

4 $$  \begin{aligned} L_{0Z_{j}}&=\sup_{H_{0Z}} L\left(k_{Z},{q_{Z1}},{q_{Z2}}|Z = Z_{j}\right)\\ &= \propto {q_{Z1}}k_{Z}^{x_{1Z}} \left(1-{q_{Z1}}k_{Z}\right)^{n_{Z}-x_{1Z}} ~ {q_{Z1}}^{X_{1}-x_{1Z}}\\ &\quad\quad\left(1-{q_{Z1}}\right)^{(N-n_{Z})-(X_{1}-x_{1Z})}\\ &\quad\times {q_{Z2}}k_{Z}^{x_{2Z}} \left(1-{q_{Z2}}k_{Z}\right)^{n_{Z}-x_{2Z}} ~ {q_{Z2}}^{X_{2}-x_{2Z}}\\ &\quad\quad(1-{q_{Z2}})^{(N-n_{Z})-(X_{2}-x_{2Z})} \;. \end{aligned}  $$

Since a closed analytical formula for $\hat {{q_{Z1}}}$, $\hat {{q_{Z2}}},\hat {k_{Z}}$ is computationally difficult to derive, we search for a numerical solution to calculate likelihood value $L_{0Z_{j}}$ and parameters estimates. We remark that differently from the univariate case, the likelihood under the null depend on *Z* and is not constant over the whole study area *G*.

To evaluate hypothesis Eq. 2 we exploit Wilks’ theorem [21] regarding procedure to test nested hypothesis.

5 $$  \lambda_{Z_{j}} = 2 \left(\ell_{Z_{j}}-\ell_{0Z_{j}}\right)  $$

which is distributed under the null hypothesis according to:

6 $$ \lambda_{Z_{j}} \overset{d}{\longrightarrow} {\chi^{2}_{1}} \;.   $$

The relative scan statistics *S* is defined as:

$$ S= \lambda_{\hat{Z}} $$

where:

$$ \hat{Z} =\lbrace Z: \lambda_{\hat{Z}} \geq \lambda_{Z_{j}} \rbrace. $$

Once $\hat {Z}$ has been identified, for potential downstream analysis it could be of interest to characterize zones by *Process*1 and *Process*2 events rate increment. This could be done by comparing the ratios $\frac {\hat {{p_{Z1}}}}{\hat {{q_{Z1}}}}$ and $\frac {\hat {{p_{Z2}}}}{\hat {{q_{Z2}}}}$ and by classifying $\hat {Z}$ as *Relative Cluster* for *Process*1 when $\frac {\hat {{p_{Z1}}}}{\hat {{q_{Z1}}}} > \frac {\hat {{p_{Z2}}}}{\hat {{q_{Z2}}}}$ and as *Relative Cluster* for *Process*2 otherwise.

**Fig. 1:**
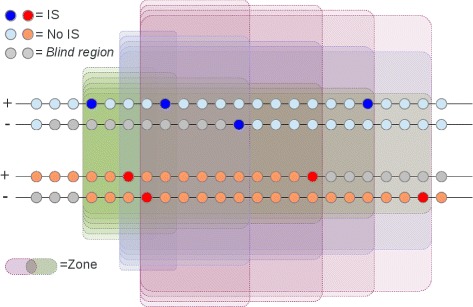
Schema of the relative scan statistics. Two data sets of Bernoulli trials are represented on an hypothetical small portion of a chromosome. *Dark blue* and *red circle*: genomic coordinate in which events (IS) was observed respectively for *DataSet*_1_ and *DataSet*_2_. *Light blue* and *orange circle*: genomic coordinates technically investigable but no-event (no integrations retrieved). *Grey circle*: *blind region* of the genome. Transparent area: example of moving windows of variable size regarding first three IS on the left

We next describe a particular property of our procedure, graphically represented in Fig. 1, that might overcome the problem of dimensionality occurring in genomic applications where the total amount Z areas can quickly approach infinity. For fixed number of successes over *Z*, namely *x*_1*Z*_ and *x*_2*Z*_, the number of failures - *n*_1*Z*_ and *n*_2*Z*_ - increases. This causes a progressive decrease of *λ*_*Z*_, until a new event occurs within the window. Since we are interested in finding $\hat {Z}$, that corresponds to the maximum *λ*_*Z*_, it is sufficient to focus on zones delimited by events (or in general success outcome).

Thus, the upper bound for the total amount of element in $\mathcal {Z}$ is [ (*X*_1_+*X*_2_)∗(*X*_1_+*X*_2_−1)/2]. Whenever is possible to define a minimum/maximum length threshold for the relative cluster, a further reduction of complexity and computational efforts holds.

The interpretation of p-value associated to relative scan statistic *S* must take into account the dimension of set $\mathcal {Z}$, corresponding to the total amount of performed tests. Since dependence between tests varies in strength and can be both positive or negative (it depends on the respective location of the zones associated to tests considered), we adopted the Holm-Bonferroni [22] method for *family wise error rate* (FWER) control. If *S* results significant, it is possible to scan the study area to identify eventual secondary significant relative cluster $\hat {Z^{*}}$ disjoint with $\hat {Z}$. For this purpose, we implement a sequential approach, thus ensuring I type error rate control and higher power [23]. The method consists in removing from *G* zone(s) previously detected as significant, redefining a new the set $\mathcal {Z^{*}}$ and values for $N_{1}^{*}$, $N_{2}^{*}$, $X_{1}^{*}$, $X_{2}^{*}$, $n_{1Z}^{*}$, $n_{2Z}^{*}$, $x_{1Z}^{*}$ and $x_{2Z}^{*}$ and sequentially performing maximization-FWER control steps.

### Algorithm

We next describe the procedure for identifying relative clusters. We designed the script for genomic binary data (e.g. viral integration data). When referring in particular to gene therapy settings, the input information needed are data sets (one data sets in univariate analysis and two data sets for multivariate comparison) relative to IS coordinates (chromosome, position and strand), *blind regions* locations if available, maximum length for candidate interesting regions, *L*_*max*_, and a minimum event counts, *EC*_*min*_. These two input parameters play a crucial role in the definition of the final output and have a strong impact on the computational effort. Their setting must be chosen carefully, according to the data sets size and computational resources available. We suggest, to avoid to exceed half of the support *G* for *L*_*max*_ (clusters greater than this threshold are not very informative) and to set *EC*_*min*_ to a small value (*EC*_*min*_≥3) in order to preserve the capability to detect possible smaller interesting regions.

A description of the algorithm in the multivariate case follows:

1. Using IS data sets and *blind regions* annotation file, calculate effective genome size *X*_1_, *X*_2_ and *N*
2. Chromosome based definition of the full set of zones, $\mathcal {Z}$.
3. Filter zones with *length*(*Z*)≥*L*_*max*_ and *EventCount*(*Z*)≤*EC*_*min*_.
4. Using IS data sets and *blind regions* annotation file, calculate effective zones size *x*_1*Z*_, *x*_2*Z*_ and *n*_*Z*_.
5. For each zone *Z*, calculate *L*_0*Z*_ (Eq. 3) and *L*_*Z*_ (Eq. 4) and corresponding *λ*_*Z*_ (Eq. 5).
6. Using ${\chi ^{2}_{1}}$ distribution, assign to each *λ*_*Z*_ a p-value (Eq. 6).
7. Apply multiple testing procedure.
8. If adjusted p-value associated to $\lambda _{\hat {Z}}$ is significant, define $G^{*}=G \setminus \hat {Z}$.
9. Calculate new $X_{1}^{*}$, $X_{2}^{*}$ and *N*^∗^ and restart from step 2.

The algorithm is implemented with a R script available upon request to the corresponding author.

## Results and discussion

### Datasets

Our application considers data sets that are comparable, for size and type of data, to those used in the literature [10] where alternative methods have been implemented to analyze and compare the profile of MLV and HIV integrations in human hematopoietic stem cells CD34+ in order to study their behaviour within the same cell type. To reduce possible technical bias the same laboratory protocol and sequencing platform was adopted.

For a detailed description of the biotechnological protocols adopted in the laboratories and subsequent bioinformatics processing steps performed, we refer to [10] and its supplementary materials. The final ISs data sets size were respectively 32631 for MLV (*X*_1_) and 28,382 for HIV (*X*_2_).

Due to various reasons related to sequencing technique (e.g. restriction enzymes) and mappability issue of the human genome (e.g. repeated sequences), the whole genome is not technically investigable. *Blind regions* are defined in the literature [17] as unobserved genomic portions which are strictly dependent on different laboratory settings and their distribution, position and total amount may change a lot across studies. However, using sophisticated and computationally intensive algorithm, it is possible to calculate and predict them quite precisely.

Regarding the univariate setting, taking into account for mappability condition allow to reduce possible systematic/technical bias and to compare clustering behaviour among experiments performed under different setting. Incorporating *blind regions* information in the multivariate scan statistics makes our approach more straightforward as compared to density estimations procedure, and their asymmetry with respect to strand does not necessary require to split analysis into two strand specific tasks. In this paper we adopt results in the literature [17] for selecting predicted *blind regions* thus reducing the genome representation to a set of *N*=4398094578 (about 2.20 ×10^9^ each strand) independent Bernoulli random variable.

A filtering procedure was applied to $\mathcal {Z}$ generated, consisting in eliminating zones longer than 2.5×10^7^ bps (considering simple difference between ISs position) and containing less then 3 ISs. This is performed in order to reduce maximization space and to focus on more biologically meaningful regions without loss of arbitrariness. The size of each zone *n*_*Z*_ is determined subtracting to the theoretical size (2 x ISs distance) the total amount (considering both strand separately) of *blind regions* contained.

### Univariate analysis results

We run single IS series analysis with scan statistics approach and we compare the results with hotspots reported in the literature [10], obtained using DBSCAN algorithm [13] (see Supplementary Material and Method in [10] for DBSCAN setting used). Some preliminary results for this analysis has been previously published in [18], without taking into account blind regions bias and focusing only on most significant findings. In HIV data set, DBSCAN identify 2446 clusters, containing 50.6 % (14,369 IS) of the total amount of IS. Clusters’ length is on average 19220 bps, but varies from a minimum of 100 to a maximum of 200500 bps. The majority (90 %) of HIV clusters are composed by 3–10 ISs.

By running univariate scan statistics methods, with a significance threshold fixed at *α*=0.01 and using Holm [22] procedure for adjusting p-values, 282 clusters are identified (see Table 1 and Additional file 1), corresponding to 45.5 % (12,935 IS) of the HIV data set. Hotposts length is between 4053 bps and 8,264,000 bps, on average 742,000, and ISs content vary from 4 to 651.

**Table 1:** List of first 10 clusters identified in HIV data by scan statistics

S	Chr	Start	End	IS count	$\frac {\hat {p_{HIV_{Z}}}}{\hat {q_{HIV_{Z}}}}$	Raw *p*-value	Adj *p*-value
2463.2	chr11	63175583	68111375	651	17.2	<2e-16	<2e-16
1795.1	chr16	95090	3640598	444	19.6	<2e-16	<2e-16
1390.0	chr17	70634094	73732441	386	15.5	<2e-16	<2e-16
1189.8	chr17	75720251	78604915	323	16.2	<2e-16	<2e-16
1063.8	chr3	46999507	52978572	424	8.5	<2e-16	<2e-16
1046.8	chr6	30563526	33532447	325	12.6	<2e-16	<2e-16
1041.8	chr9	138245676	139772487	224	26.9	<2e-16	<2e-16
732.0	chr8	144469820	146194757	188	18.1	<2e-16	<2e-16
721.1	chr19	572963	3118599	209	14.3	<2e-16	<2e-16
629.1	chr17	1483915	4578114	238	9.2	<2e-16	<2e-16

For MLV, DBSCAN identifies 3497 clusters, corresponding to 65.3 % (21,307 IS) of MLV data set. Clusters are on average 8385 bps long, the observed minimum and maximum length are respectively 19 bps and 78,530 bps. Using univariate scan statistics, 803 clusters has been identified (see Table 2 and Additional file 1), grouping 18,388 ISs equivalent to 56.3 % of MLV data set. Length mean value results equal to 270,400 bps, with a minimum of 1932 bps and a maximum of 5,449,000 bps.

**Table 2:** List of first 10 clusters identified in MLV data by scan statistics

S	Chr	Start	End	IS count	$\frac {\hat {p_{MLV_{Z}}}}{\hat {q_{MLV_{Z}}}}$	Raw *p*-value	Adj *p*-value
386.5	chr20	51646845	51991770	89	22.8	<2E-16	<2E-16
326.4	chr20	10362242	10450134	55	51.8	<2E-16	<2E-16
318.4	chr17	26646082	26672265	41	131.1	<2E-16	<2E-16
302.6	chr17	76325116	76460372	56	39.5	<2E-16	<2E-16
285.6	chr19	59566413	59591310	37	127.9	<2E-16	<2E-16
284.6	chr21	38671040	39311896	90	12.2	<2E-16	<2E-16
279.2	chr17	51718847	53782415	142	6.2	<2E-16	<2E-16
278.7	chr1	25046795	28847012	183	4.7	<2E-16	<2E-16
267.7	chr18	72291047	72971441	87	11.6	<2E-16	<2E-16
264.4	chr12	6084417	10441567	197	4.2	<2E-16	<2E-16

**Fig. 2:**
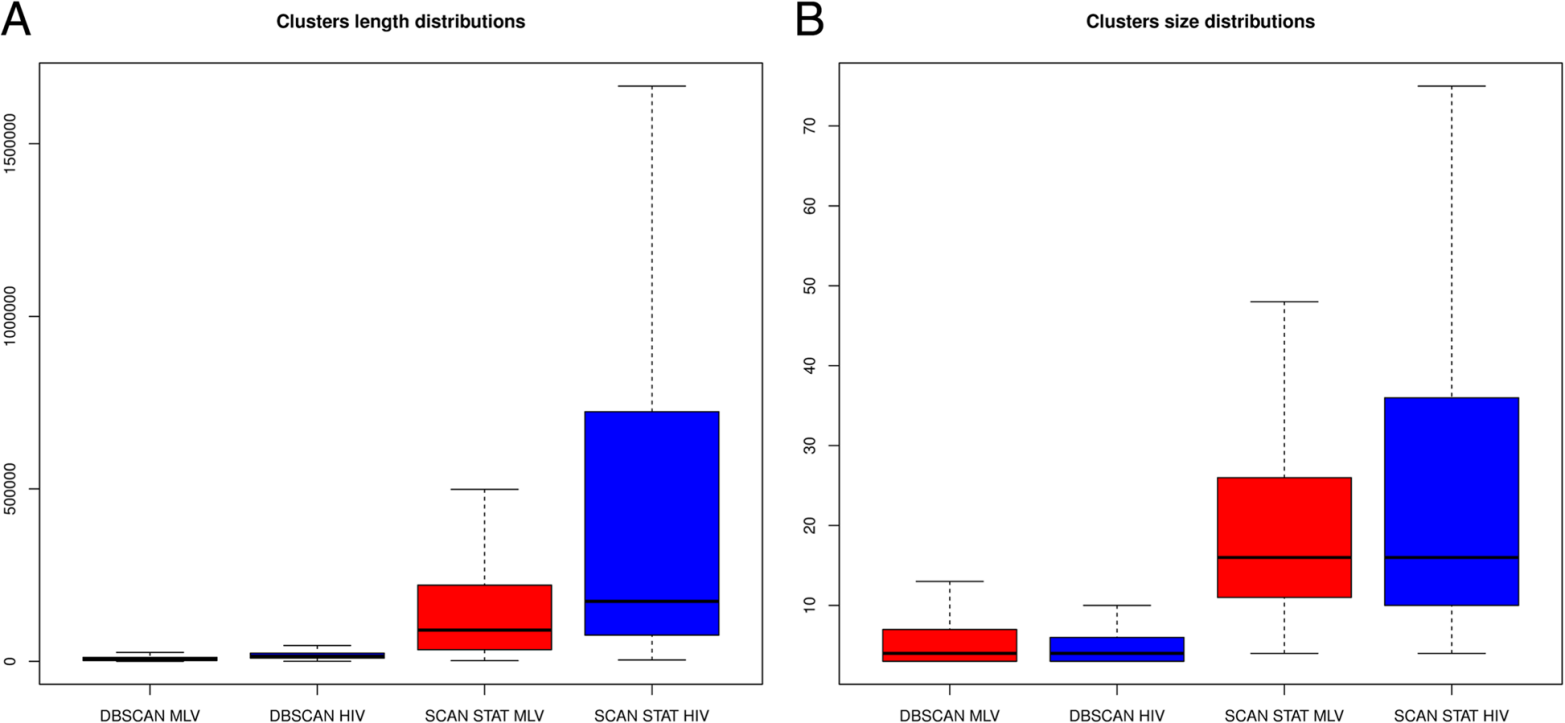
**a** Length distributions of clusters identified by DBSCAN and scan statistics algorithm in MLV and HIV data sets. **b** Size distributions of clusters identified by DBSCAN and scan statistics algorithm in MLV and HIV data sets

In general, the two methods provide consistent results and highlight different clustering behaviour proper of the two viral vectors, in particular in terms of clusters length and events density. Both methods confirm HIV preference for active transcriptional units, such as coding regions, typically wider than regulatory regions preferentially targeted by MLV viral vectors. This characteristic is well captured in particular by the success probability ratio, $\frac {\hat {p_{HIV_{Z}}}}{\hat {q_{HIV_{Z}}}}$ for HIV candidate hotspots, generally lower with respect to MLV counterpart, $\frac {\hat {p_{MLV_{Z}}}}{\hat {q_{{Z}}}}$ (see Tables 1, 2 and Additional files 1 and 2). The count distributions of ISs belonging to the same cluster are similar across virus type but not across methods. Taking into account summary data and graph in Fig. 2, is clear that DBSCAN lead to a bigger selection of over targeted regions than scan statistics, characterized by both smaller length and size. We remark that both methods suggest a clear difference in terms of length between vectors type and a homogeneity for size distributions, reinforcing the findings known about virus preferences.

We next investigate how methods agree in identifying locations of most significant regions. DBSCAN clusters are sorted in terms of size, i.e. the amount of IS falling within cluster limits, to allow for possible the comparison with scan statistics results.

The list of the first 10 Most Significant Clusters (MSCs) coordinates discovered by Scan Statistics in HIV data set are showed in Table 1, together with some related measures. The complete list is available in Additional file 1. The most significant cluster is located at chromosome 11, interval 63,175,583;68,111,375 and within the same region DBSCAN identifies 40 out of 2446 distinct clusters, including the top 2 for ISs content (interval 65,586,752;65,736,062, 110 ISs and interval 66651503-66776194, 96 ISs). The second most significant cluster, named *MSC*_2_ is located on chromosome 16, interval 71,294,851;77,821,445 and is composed by 610 IS. Within this genomic region, DBSCAN reported 38 clusters, including the third in terms of ISs.

Univariate analysis results for MLV data set are tabulated in Table 2 and Additional file 2. Region on chromosome 20, interval 51,646,845;51,991,770 contain 89 ISs and is suggested to be the most evident hotspot region for MLV vector. Within the same interval, DBSCAN identify 8 distinct clusters, but not among the top in ranking. The second, *MSC*_2_,is on chromosome 20, interval 10,362,242;10,450,134 and is composed by 55 ISs. It overlaps with the 50-th hotspost retrieved using DBSCAN. A perfect correspondence is observed between *MSC*_3_ and the 4-th cluster derived from DBSCAN, both located on chromosome 17, interval 26,659,383;26,672,265. Conversely, the first cluster calculated using DBSCAN is on chromosome 22 27,525,356;27,545,150, its size is 42 ISs and corresponds to 85-th MLV scan statistics derived cluster.

In simple terms we reveal that the most important part of the difference in identifying the total amount of clusters can be attributed to a *fragmentation* of scan statistics cluster in more DBSCAN clusters. Despite that, an overall clear correspondence in terms of localization was observed, while agreement in ranking is more dependent on clustering behaviour.

### Multivariate analysis results

**Fig. 3:**
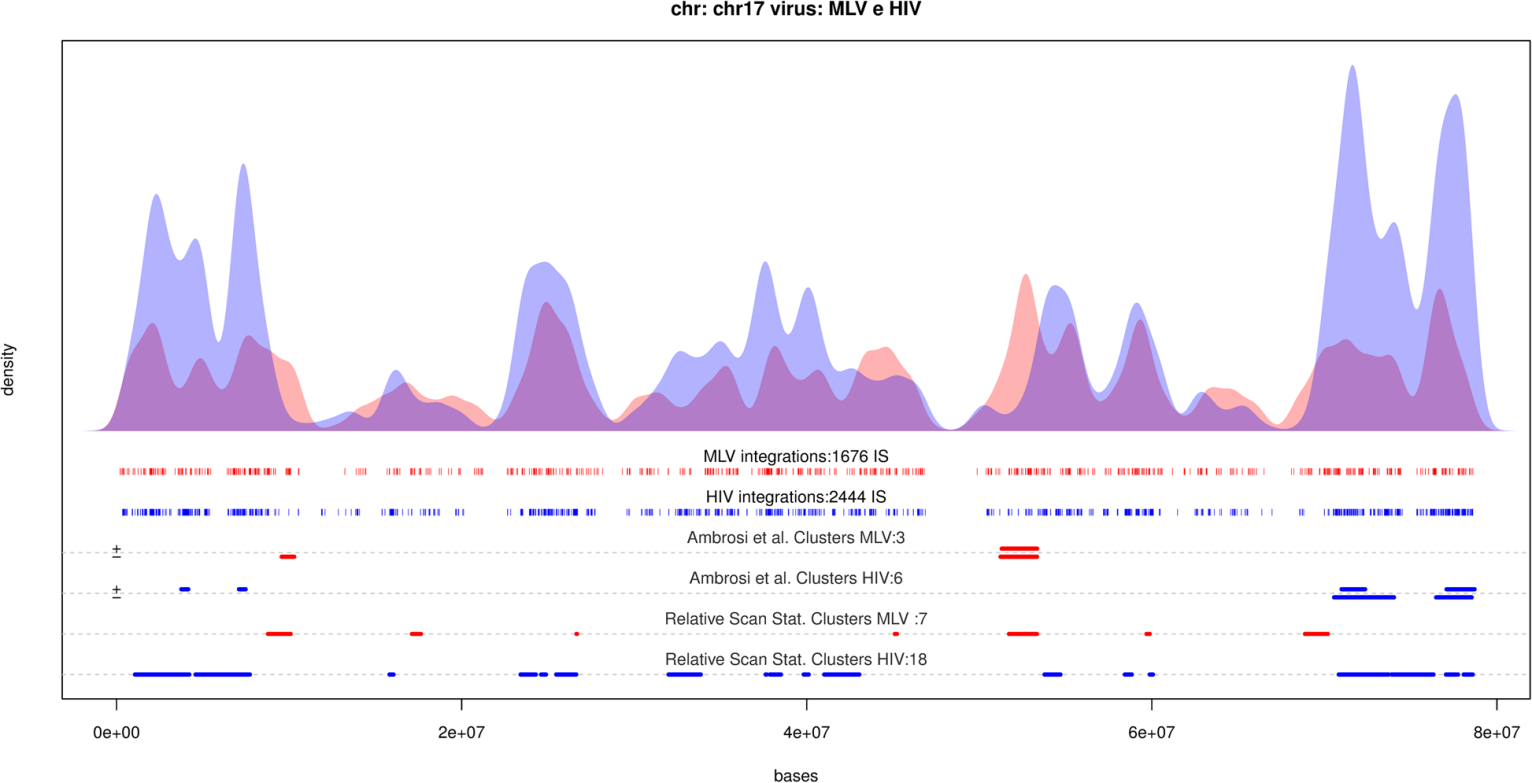
HIV and MLV IS distributions on chr 17. HIV and MLV IS distributions on chromosome 17 estimated by means of Gaussian kernel with unbiased cross validation bandwidth selection (*blue curve* and *red curve* respectively). Comparative hotspots reported in [17] correspondent to segments indicated on third line in red (MLV comparative hotspot) and fourth line in blue (HIV comparative hotspots) taking into account for strand annotation. Fifth and sixth lines are dedicated to relative scan statistics. First two significant cluster identified using relative scan statistics with no correspondent comparative hotspots are highlighted (*black box*)

The Relative Scan Statistics identified 292 genomic intervals showing a difference in targeting propensity by the two viral vectors. Totally, 174 of them could be classify as relative clusters for MLV. Conversely 119 of them are labeled as HIV relative clusters. Chromosome 17 is the one with the highest amount of detected interesting regions (Fig. 3).

We remark that the a big advantage of the proposed methods is the ability to detect both long and short regions. Long relative cluster can be usually easily visualized by using density estimate superposition. Short relative clusters or closed *opposite* relative cluster are much more difficult to detect, due to the smoothness of kernel estimator. This is in our opinion a crucial feature of our proposal, and it may be of particular utility for data analysis and for vector safety assessment. We now compare our list with the suggested 100 regions (51 for MLV and 49 for HIV) proposed in the literature [17].

Although the total amount of interesting regions might vary considerably, it is not clear which one performs better since true differently targeted regions are not known. In our opinion, since the underlying biological mechanism and target site selection process are deeply different (MLV belongs to the gammaretroviral genus and HIV to the lentiviral), a longer list of candidate regions can be considered more realistic.

**Fig. 4:**
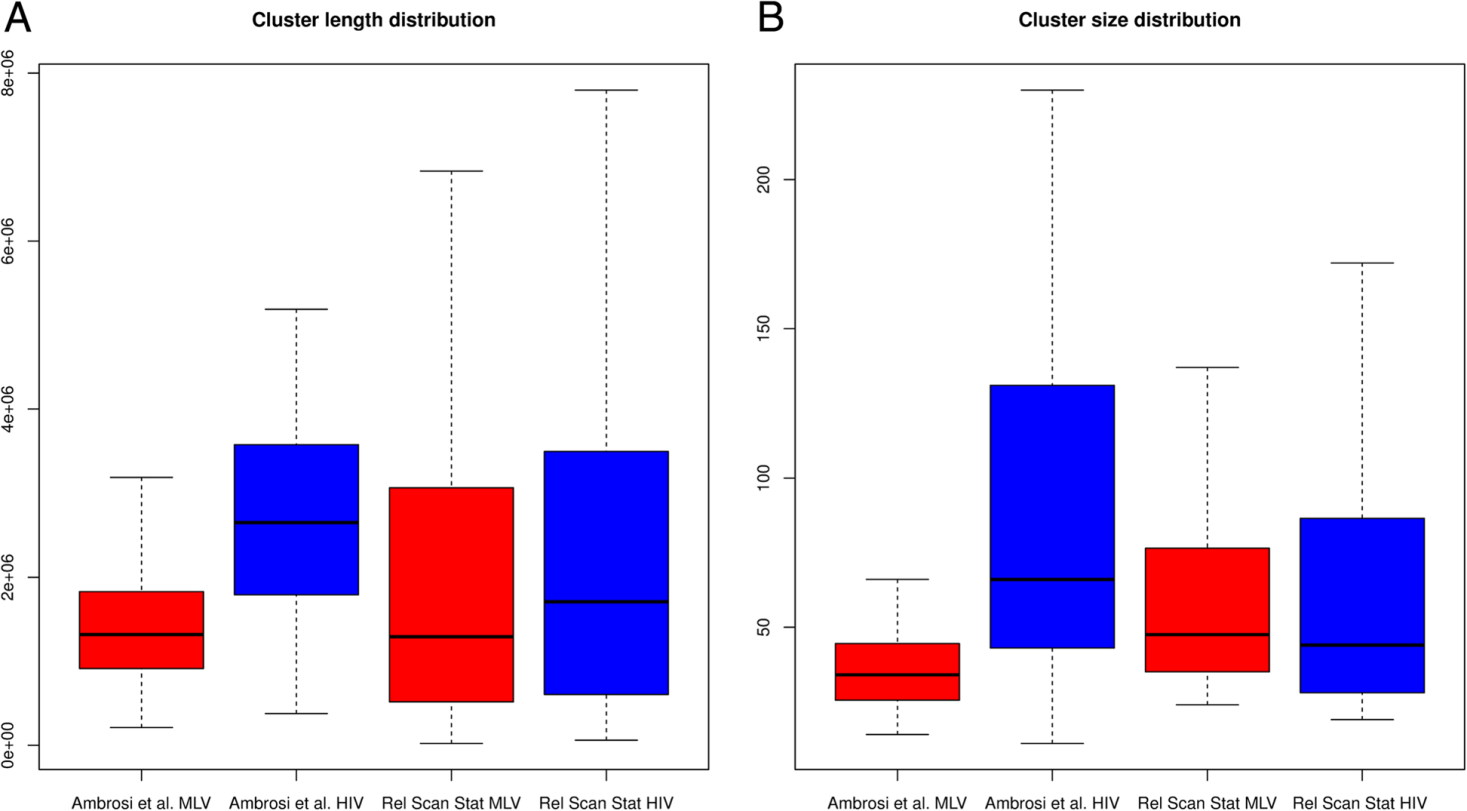
**a** Length distributions of clusters identified by Ambrosi et al. methods and relative scan statistics algorithm in MLV and HIV data sets. **b** Size distributions of clusters identified by Ambrosi et al. methods and relative scan statistics algorithm in MLV and HIV data sets

This idea seems to be supported by visual comparison of chromosome based kernel density estimations. The length and the size of regions identified using the two different approach are similar (Fig. 4), nevertheless [17] method discriminates between MLV and HIV regions, since the latter are longer and include more events. By comparing intervals localization and their overlapping, we can highlight that all previously identified regions are associated to a Relative Scan Statistics derived clusters.

**Fig. 5:**
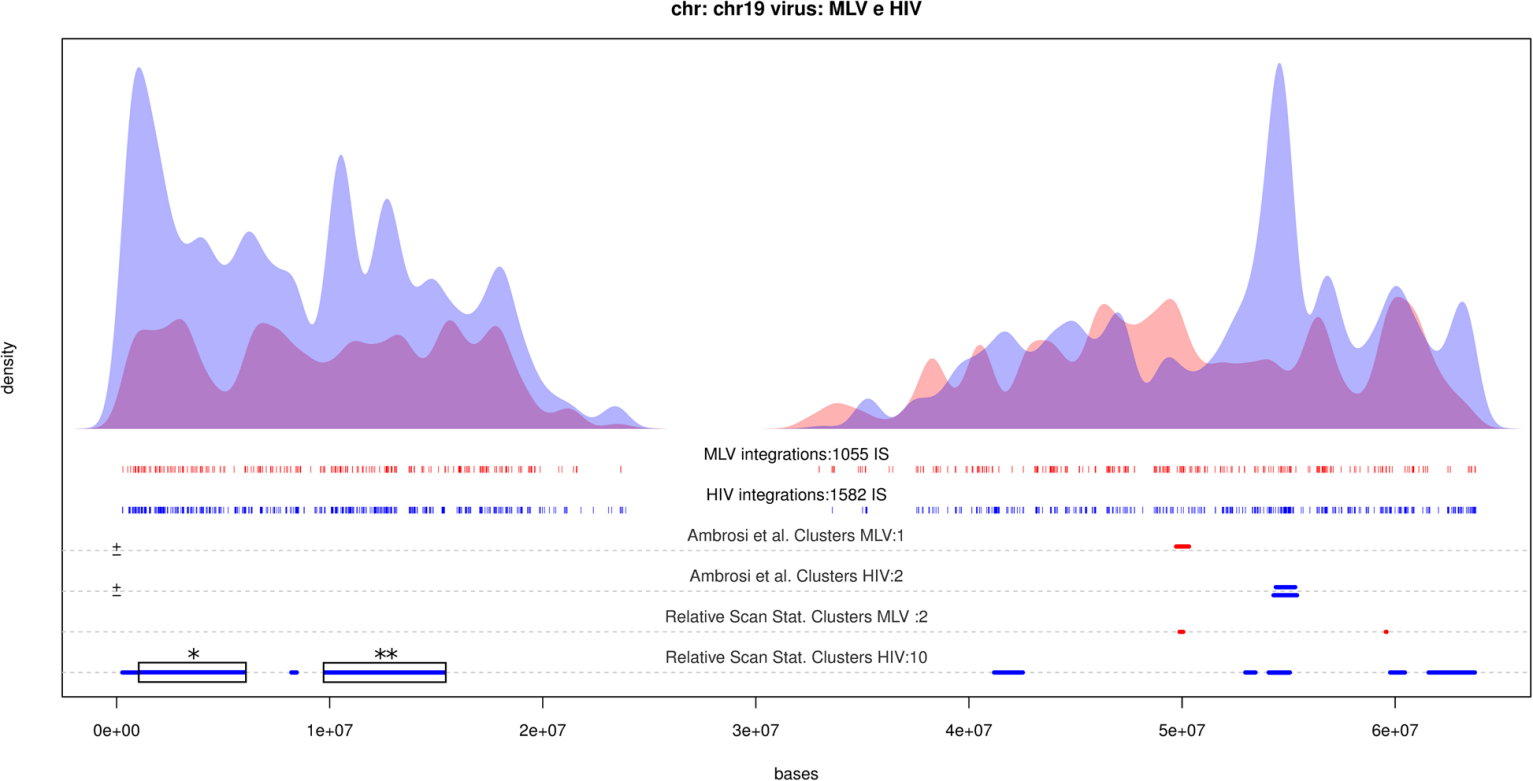
HIV and MLV IS distributions on chr 19. HIV and MLV IS distributions on chromosome 19 estimated by means of Gaussian kernel with unbiased cross validation bandwidth selection (*blue curve* and *red curve* respectively). Comparative hotspots reported in [17] correspondent to segments indicated on third line in red (MLV comparative hotspot) and fourth line in blue (HIV comparative hotspots) taking into account for strand annotation. Fifth and sixth lines are dedicated to relative scan statistics. First two significant cluster identified using relative scan statistics with no correspondent comparative hotspots are highlighted (*black box*)

In Table 3 first 20 relative clusters are reported (complete list available as Additional file 3). For both methods, the most significant regions are labeled as cluster for HIV vector, suggesting that it is easier to detect wider regions characterized by moderate increase of targeting rate, typical of HIV vector, than shorter genomic portions with high increase of targeting probability as observed for MLV derived vector. To compare also the ranking of regions, we sorted the results obtained in [17] using p-value associated to Fisher exact test calculated for assess regions significance. Due to strand specificity, top 6 results in reported in [17] map to the top 3 regions in Table 3. However the known method missed the firsts 2 regions both located on chromosome 19 on p-arm, Fig. 5 which is a gene dense portions of the genome. Gene density is known to be a particular feature in the genome able to attract particularly HIV derived vectors and this support our result.

**Table 3:** List of relative clusters identified by relative scan statistics

S	Chr	Start	End	HIV IS	MLV IS	$\log \left (\frac {\frac {\hat {p_{HIV_{Z}}}}{\hat {q_{HIV_{Z}}}}}{\frac {\hat {p_{MLV_{Z}}}}{\hat {q_{MLV_{Z}}}}}\right)$	Type	Adj *p*-value
474.1	chr11	63153734	68347426	659	129	1.91	hiv	<2E-16
450.9	chr6	30095760	33488528	332	7	4.49	hiv	<2E-16
434.2	chr16	95090	3561021	430	41	2.74	hiv	<2E-16
260.9	chr17	70835415	73732441	372	75	1.86	hiv	<2E-16
227.0	chr3	47041751	52978572	422	119	1.47	hiv	<2E-16
219.4	chr9	134493480	139818935	307	60	1.89	hiv	<2E-16
213.5	chr17	77047796	77746204	172	7	3.70	hiv	<2E-16
191.9	chr8	144548769	146194757	182	15	2.89	hiv	<2E-16
122.0	chr19	1027304	6006371	292	104	1.20	hiv	<2E-16
115.4	chr22	48983597	49573459	115	11	2.71	hiv	<2E-16
105.6	chr21	37559632	39311896	9	126	-3.02	mlv	<2E-16
102.1	chr19	54074745	55048471	122	18	2.21	hiv	<2E-16
99.3	chr17	1069411	4213267	229	79	1.23	hiv	<2E-16
96.4	chr1	153550587	154168170	90	7	2.94	hiv	<2E-16
91.8	chr18	70832211	73059134	6	103	-3.26	mlv	<2E-16
91.5	chr17	4573721	7723628	194	62	1.32	hiv	<2E-16
86.5	chr20	49745347	52129713	7	102	-3.07	mlv	<2E-16
86.0	chr12	11729500	14430150	8	105	-2.95	mlv	<2E-16
83.3	chr20	60901158	62379063	109	19	2.02	hiv	<2E-16
81.3	chr6	6536008	13289623	22	141	-2.13	mlv	<2E-16

Graphs for remaining chromosomes are available in Additional file 4.

## Conclusions

In this paper we present two methods for clustering identification of genomic events based on scan statistics approach. Results retrieved from both methods are consistent with the biological literature and findings thus revealing deep biological differences between integration process and target sites selection characterizing different viral vectors. Speculating on cluster dimensions and length, our analysis confirms the well known preferences of MLV in integrating more likely in regulatory elements or in general over small genomic interval, whereas HIV integrates over wider regions corresponding to active coding elements. Independently from the total amount of identified interesting regions, a substantial spatial overlap between results was observed in HIV data set, as regarding both localization and significance. For MLV data set, a good agreement is showed in terms of localization but for significance ranking. The intrinsic behaviour of HIV probably helps this results correspondence, since aggregation is less strong than MLV but affects wider regions, leading to cluster formed by many IS rewarded by DBSCAN ranking scheme based on dimension. For MLV instead, generally the aggregation tendency is characterized by higher event density but limited to narrow genomic intervals and less ISs.

Relative Scan Statistics seems to be able to identify regions characterized by unshared variation of events rate, potentially allowing for focusing downstream analysis only on differently targeted regions. This may help clinicians/researcher in improve viral vectors safety. The results obtained agree with previous published literature and avoid the necessity to split analysis according to strands.

In conclusion, starting from a probabilistic approach based on estimation and comparison of probability of success, we recommended scan statistics as a fundamental inferential tool able to exploit an hypothesis testing procedure to sort candidate regions in terms of significance instead of size or additional testing procedure.

## Additional files

Additional file 1
**Table S1.** Full list of HIV clusters. (PDF 49 kb)

Additional file 2
**Table S2.** Full list of MLV clusters. (PDF 80 kb)

Additional file 3
**Table S3.** Full list of relative clusters. (PDF 51 kb)

Additional file 4
**Figure S1.** Relative scan statistics results for remaining chromosomes. (PDF 5089 kb)
