# Supplementary material for: A novel scan statistics approach for clustering identification and comparison in binary genomic data
Source: BMC Bioinformatics. 2016 Sep 22;17(Suppl 11):320. doi: 10.1186/s12859-016-1173-8 (PMC5046198; doi:10.1186/s12859-016-1173-8)
Supplement: Additional file 1 — Table S1. Full list of HIV clusters. (PDF 49 kb) [file 12859_2016_1173_MOESM1_ESM.pdf]

Table 1: List of clusters identified in HIV data by Scan Statistics.

S	Chr	Start	End	IS count	$\frac{p_{H\hat{I}V_Z}}{q_{H\hat{I}V_Z}}$	Raw pvalue	Adj pvalue
2463.2	chr11	63175583	68111375	651	17.2	<2e-16	<2e-16
1795.1	chr16	95090	3640598	444	19.6	<2e-16	<2e-16
1390.0	chr17	70634094	73732441	386	15.5	<2e-16	<2e-16
1189.8	chr17	75720251	78604915	323	16.2	<2e-16	<2e-16
1063.8	chr3	46999507	52978572	424	8.5	<2e-16	<2e-16
1046.8	chr6	30563526	33532447	325	12.6	<2e-16	<2e-16
1041.8	chr9	138245676	139772487	224	26.9	<2e-16	<2e-16
732.0	chr8	144469820	146194757	188	18.1	<2e-16	<2e-16
721.1	chr19	572963	3118599	209	14.3	<2e-16	<2e-16
629.1	chr17	1483915	4578114	238	9.2	<2e-16	<2e-16
624.3	chr22	48882830	49493771	123	33.4	<2e-16	<2e-16
596.8	chr17	6770502	8437986	180	13.3	<2e-16	<2e-16
562.6	chr16	87165726	88407235	144	18.2	<2e-16	<2e-16
538.1	chr19	53299175	55229231	170	12.2	<2e-16	<2e-16
450.0	chr17	23916908	26720712	197	7.5	<2e-16	<2e-16
412.7	chr9	129569084	135137989	239	5.4	<2e-16	<2e-16
397.9	chr17	37619828	40732172	182	7.1	<2e-16	<2e-16
382.3	chr16	29455822	31233731	140	9.6	<2e-16	<2e-16
350.8	chr1	148320146	154758622	255	4.3	<2e-16	<2e-16
342.8	chr20	61696801	62379063	84	19.9	<2e-16	<2e-16
335.9	chr12	6310112	10134633	193	5.4	<2e-16	<2e-16
326.1	chrX	152815196	153697523	95	14.1	<2e-16	<2e-16
322.3	chr19	3908200	6364236	138	7.7	<2e-16	<2e-16
319.7	chr12	51689586	56423768	208	4.8	<2e-16	<2e-16
311.6	chr19	10069817	12591618	144	7.0	<2e-16	<2e-16
307.0	chr11	71207956	73591922	146	6.7	<2e-16	<2e-16
287.2	chr1	734823	1800605	85	13.7	<2e-16	<2e-16
286.4	chr11	62086312	62398734	64	24.5	<2e-16	<2e-16
278.0	chr12	47332987	50000471	147	5.9	<2e-16	<2e-16
277.4	chr16	65133795	68293674	155	5.6	<2e-16	<2e-16
244.6	chr1	21008023	29272246	245	3.3	<2e-16	<2e-16
242.1	chr17	53405986	55948631	136	5.5	<2e-16	<2e-16
234.9	chr15	38241912	41328190	144	5.0	<2e-16	<2e-16
229.1	chr5	176293705	179927141	149	4.8	<2e-16	<2e-16
222.0	chr2	27110680	28321038	93	7.9	<2e-16	<2e-16
209.9	chr19	12591618	14731512	105	6.3	<2e-16	<2e-16
198.3	chr22	38787587	40607337	102	6.1	<2e-16	<2e-16
188.6	chr11	208639	932165	56	13.6	<2e-16	<2e-16
182.9	chr12	119068070	122789258	135	4.2	<2e-16	<2e-16
179.6	chr17	58437063	60085404	93	6.1	<2e-16	<2e-16
164.9	chr4	677564	3115594	95	5.4	<2e-16	<2e-16
162.8	chr12	131713150	131777187	27	54.4	<2e-16	<2e-16
159.1	chr19	16052764	18545718	95	5.2	<2e-16	<2e-16
150.8	chr19	56416749	56427932	18	178.0	<2e-16	<2e-16
150.3	chr19	59308436	60890393	74	6.4	<2e-16	<2e-16
146.2	chr19	6737355	8543708	80	5.7	<2e-16	<2e-16
140.1	chr17	74189332	74345669	33	21.7	<2e-16	<2e-16
135.3	chr17	32954318	36054933	105	4.0	<2e-16	<2e-16
134.1	chr7	99336741	100320713	58	7.6	<2e-16	<2e-16
131.9	chr11	46031066	47813396	82	5.0	<2e-16	<2e-16
124.6	chr21	46705611	46888293	32	18.0	<2e-16	<2e-16
124.2	chr17	5099510	5257613	31	19.1	<2e-16	<2e-16
119.0	chr1	8442685	12465479	118	3.3	<2e-16	<2e-16
118.6	chr15	72534214	74126803	71	5.2	<2e-16	<2e-16
116.1	chr16	14983562	16136909	63	5.7	<2e-16	<2e-16
111.3	chr1	42460569	46187449	114	3.3	<2e-16	<2e-16
110.1	chr19	46754455	46765612	14	137.6	<2e-16	<2e-16
109.4	chr12	260290	1972238	75	4.5	<2e-16	<2e-16
108.1	chr6	34367632	37375345	100	3.5	<2e-16	<2e-16
106.5	chr20	2797532	2897527	24	24.0	<2e-16	<2e-16
106.4	chr15	89051854	89206145	27	18.5	<2e-16	<2e-16
94.2	chr6	42659593	43688345	52	5.6	<2e-16	<2e-16
92.7	chr1	19327528	19412219	21	23.7	<2e-16	<2e-16
91.9	chr19	43835529	43871431	16	47.0	<2e-16	<2e-16
90.1	chr19	62455985	63788372	57	4.9	<2e-16	<2e-16
90.1	chr16	18702640	19463940	46	6.2	<2e-16	<2e-16
88.6	chr7	2187088	2819386	37	7.9	<2e-16	<2e-16
86.1	chr20	60996341	61050308	17	33.2	<2e-16	<2e-16
84.8	chr19	56708061	56780524	18	27.7	<2e-16	<2e-16
83.3	chr16	3640598	3861615	26	12.5	<2e-16	<2e-16
82.9	chr1	36185064	36251028	18	26.2	<2e-16	<2e-16
81.4	chr21	44114701	45458600	50	5.0	<2e-16	<2e-16
80.0	chr20	32445317	33971001	60	4.2	<2e-16	<2e-16
77.7	chr17	15867404	16072788	25	11.8	<2e-16	<2e-16
72.2	chr22	37091774	37485352	29	8.4	<2e-16	<2e-16
71.2	chr9	127552555	127645770	18	18.6	<2e-16	<2e-16
69.7	chr22	28617146	28891106	26	9.3	1.11E-016	8.49E-013
69.2	chr7	5641305	5780795	20	14.3	1.11E-016	6.03E-012
68.9	chr14	102879554	106316542	78	3.0	1.11E-016	3.01E-012
66.7	chr4	151444378	151953092	33	6.4	3.33E-016	1.93E-011
65.8	chr14	22443920	23032540	33	6.3	4.44E-016	9.91E-012
64.8	chr17	23416076	23547671	19	13.9	7.77E-016	7.96E-012
64.6	chr11	58071143	58123998	14	26.3	8.88E-016	2.78E-011
63.8	chr12	131007062	131188639	20	12.3	1.44E-015	4.54E-011
62.6	chr10	73950559	75964612	65	3.2	2.55E-015	7.08E-011
60.4	chr12	46341616	46505124	18	13.5	7.55E-015	2.35E-010

59.9	chr11	9346783	10234648	41	4.5	1.02E-014	3.12E-010
59.7	chr11	61874307	61901951	11	40.0	1.09E-014	2.95E-010
58.8	chr2	213557948	213717466	19	11.7	1.74E-014	1.71E-009
58.8	chr19	40833483	41421015	29	6.4	1.77E-014	1.70E-010
58.7	chr19	51881530	52376490	28	6.7	1.87E-014	1.41E-010
58.5	chr11	116292822	118657236	66	3.1	2.02E-014	5.37E-010
58.3	chr9	124812213	124877771	14	20.8	2.26E-014	5.64E-010
57.4	chr10	103540391	104947931	49	3.7	3.59E-014	8.20E-010
57.0	chr1	204413594	204417647	7	158.7	4.29E-014	3.43E-009
55.7	chr17	42551392	45248142	71	2.8	8.43E-014	8.45E-010
55.7	chr9	137862339	138059547	18	11.7	8.58E-014	1.96E-009
55.7	chr2	188015551	188061707	12	26.6	8.59E-014	8.19E-009
55.2	chr17	41493506	41644173	18	11.6	1.08E-013	6.04E-010
54.2	chr19	44522314	44649484	15	15.5	1.82E-013	1.13E-009
52.4	chr8	125386634	125608196	20	9.0	4.47E-013	1.35E-008
52.1	chr1	16050762	16165426	15	14.4	5.37E-013	4.11E-008
51.6	chr5	416495	467707	11	27.4	6.73E-013	3.25E-008
51.4	chr11	28013476	28208831	19	9.5	7.66E-013	1.70E-008
51.1	chr3	196476899	199205493	68	2.8	8.80E-013	6.36E-008
51.0	chr9	114032053	114203149	18	10.2	9.26E-013	2.08E-008
50.6	chr6	109414949	109590913	18	10.0	1.14E-012	5.65E-008
50.4	chr22	40910583	41901310	35	4.5	1.27E-012	7.43E-009
50.2	chr16	28058263	29028848	36	4.3	1.35E-012	1.46E-008
49.7	chr2	241864851	242021367	17	10.7	1.79E-012	1.62E-007
49.7	chr20	3762179	3938065	17	10.7	1.82E-012	9.81E-009
49.0	chr17	344837	1244244	33	4.6	2.50E-012	1.34E-008
49.0	chr19	3313758	3548859	16	11.5	2.61E-012	1.38E-008
48.4	chr12	22520558	22735200	19	8.7	3.42E-012	1.02E-007
48.1	chr12	114936760	115151065	19	8.6	3.98E-012	1.12E-007
47.4	chr15	87435869	88376030	36	4.1	5.79E-012	8.42E-008
47.3	chr17	4758828	4824767	11	22.4	5.94E-012	2.91E-008
46.8	chr6	76390254	76438927	11	21.8	7.72E-012	3.64E-007
46.3	chr1	52658945	52724180	12	17.7	1.00E-011	7.59E-007
46.1	chr15	91231839	91241368	7	72.1	1.12E-011	1.51E-007
46.0	chr3	16913674	18458859	50	3.1	1.19E-011	8.17E-007
45.2	chr19	39357722	39395984	10	25.0	1.79E-011	9.16E-008
45.2	chr4	35773527	35903236	15	11.2	1.80E-011	9.70E-007
44.5	chr16	51743121	51854705	14	12.3	2.59E-011	2.58E-007
44.3	chrX	46279876	47692480	45	3.3	2.81E-011	5.33E-007
44.3	chr16	11688564	11906253	18	8.2	2.83E-011	2.55E-007
43.9	chr8	90834007	90875983	10	23.4	3.51E-011	9.81E-007
43.6	chr22	43921384	43950282	9	29.6	4.03E-011	2.03E-007
43.5	chr7	44415873	45072724	28	4.8	4.15E-011	2.13E-006
42.6	chr7	7222556	7254855	9	28.0	6.57E-011	3.19E-006
42.6	chr2	124262023	124430777	16	9.2	6.78E-011	6.05E-006
42.3	chr12	14434677	14514042	12	14.8	7.92E-011	2.12E-006
42.1	chr6	99964634	100064527	13	12.7	8.74E-011	3.95E-006
42.0	chr15	32330526	32407715	12	14.6	9.00E-011	1.18E-006
42.0	chr2	81752840	81765392	7	53.4	9.35E-011	8.18E-006
41.8	chr4	83978775	84103005	14	11.1	1.01E-010	5.22E-006
41.6	chr12	108819352	109481248	29	4.4	1.12E-010	2.90E-006
41.5	chr6	45395081	45430023	9	26.3	1.16E-010	5.09E-006
41.3	chr5	100168498	100249968	12	14.1	1.33E-010	6.37E-006
40.9	chr8	22365049	22446743	12	13.9	1.64E-010	4.32E-006
40.6	chr12	68933062	69018816	12	13.7	1.90E-010	4.45E-006
40.3	chr17	46436203	46502688	11	16.0	2.17E-010	1.05E-006
40.1	chr15	61597299	63885304	58	2.6	2.39E-010	3.03E-006
39.8	chr6	26235735	26344272	13	11.5	2.79E-010	1.17E-005
39.5	chr3	185924587	186183651	18	7.1	3.25E-010	2.01E-005
39.4	chr9	35453944	36098692	27	4.5	3.38E-010	7.16E-006
39.0	chr17	32580339	32770599	16	8.1	4.23E-010	1.96E-006
38.9	chr15	97961864	98032923	11	14.9	4.41E-010	3.78E-006
38.9	chr10	89616181	89731882	13	11.1	4.51E-010	8.68E-006
38.8	chr6	13730899	13804728	11	14.8	4.76E-010	1.89E-005
38.5	chr15	56706219	56997606	19	6.4	5.46E-010	4.42E-006
38.3	chr19	14863079	15385295	23	5.2	5.93E-010	2.73E-006
38.2	chr10	112619306	112717544	12	12.3	6.33E-010	1.12E-005
38.0	chr5	138641804	138797952	14	9.5	7.13E-010	3.28E-005
37.9	chr12	60980678	61076729	12	12.2	7.27E-010	1.57E-005
37.9	chr6	135771532	135799691	8	28.0	7.57E-010	2.92E-005
37.8	chr7	73155325	73805539	25	4.7	7.67E-010	3.63E-005
37.8	chr8	133841083	133918764	11	14.1	7.92E-010	2.00E-005
37.6	chr12	63858620	63917380	10	16.8	8.48E-010	1.68E-005
37.6	chr14	70459470	70605266	14	9.3	8.78E-010	1.61E-005
37.6	chr19	35153756	35212130	10	16.8	8.79E-010	3.95E-006
37.4	chr20	45343343	45695323	20	5.8	9.73E-010	4.69E-006
37.0	chr22	20469392	20528768	10	16.3	1.15E-009	5.26E-006
37.0	chr22	29859225	30537775	27	4.3	1.19E-009	4.89E-006
36.9	chr6	124195805	124852286	28	4.1	1.25E-009	4.64E-005
36.8	chr22	48584223	48656424	10	16.1	1.29E-009	4.45E-006
36.6	chr1	143924593	144004072	11	13.3	1.45E-009	1.07E-004
36.6	chr2	32522975	32674360	14	9.0	1.46E-009	1.27E-004
36.6	chr3	42574218	42659580	11	13.3	1.48E-009	8.79E-005
36.5	chr11	95705778	95749757	9	19.7	1.50E-009	3.11E-005
36.5	chr16	54987392	57190838	52	2.6	1.54E-009	1.24E-005
35.9	chr9	20342445	20921478	26	4.3	2.04E-009	3.81E-005
35.6	chr22	22744389	23213509	21	5.2	2.42E-009	8.12E-006
35.5	chr11	60791121	60973345	14	8.6	2.53E-009	4.94E-005
35.5	chr4	81062184	81277735	16	7.2	2.55E-009	1.24E-004
34.8	chr2	162857980	162927400	10	14.5	3.61E-009	3.02E-004
34.7	chr6	147537722	147733166	15	7.6	3.78E-009	1.25E-004

34.7	chr16	86552295	86612161	9	17.7	3.78E-009	2.11E-005
34.7	chr9	3256865	3513377	17	6.5	3.81E-009	6.39E-005
34.7	chr17	27296192	27730675	21	5.1	3.91E-009	1.57E-005
34.5	chr5	162784146	162874789	11	12.0	4.28E-009	1.87E-004
34.3	chr19	46467290	46585259	11	11.9	4.73E-009	2.08E-005
34.2	chr2	61357441	61407274	9	17.1	5.03E-009	4.04E-004
34.1	chr22	44473967	45116916	23	4.6	5.22E-009	1.46E-005
34.1	chr11	82550591	82664547	12	10.2	5.28E-009	9.97E-005
33.6	chr7	79606232	79676518	10	13.5	6.86E-009	3.07E-004
33.6	chr13	114037709	114062164	7	28.8	6.93E-009	1.24E-004
33.5	chr14	44488274	44723055	16	6.7	7.03E-009	1.17E-004
33.4	chr1	159292487	159590875	17	6.2	7.51E-009	5.54E-004
33.1	chr7	130279553	130825064	24	4.3	8.53E-009	3.58E-004
33.1	chr7	123118632	123172725	9	16.1	8.56E-009	3.38E-004
32.7	chr22	34495851	35197090	26	4.0	1.10E-008	2.57E-005
32.5	chr3	28305246	28362154	9	15.5	1.19E-008	6.82E-004
32.4	chr19	18808218	19421217	21	4.8	1.23E-008	4.87E-005
32.2	chr16	52210437	52313581	11	10.7	1.40E-008	7.21E-005
32.1	chr9	26831312	27045312	15	6.8	1.49E-008	2.27E-004
32.0	chr19	43133388	43212852	10	12.4	1.53E-008	5.28E-005
32.0	chr3	25623173	25781338	13	8.2	1.55E-008	8.52E-004
31.9	chr17	57192569	57472069	17	5.9	1.58E-008	5.68E-005
31.5	chr11	1877860	1885461	5	62.5	1.98E-008	3.55E-004
31.5	chr9	79008931	79166455	13	8.1	2.02E-008	2.91E-004
31.5	chr12	93224250	93352836	12	9.0	2.03E-008	3.69E-004
31.4	chr1	98060708	98122797	9	14.5	2.09E-008	1.49E-003
31.3	chr12	25120003	25148288	7	24.4	2.21E-008	3.69E-004
31.2	chr17	70209114	70274344	8	18.0	2.36E-008	7.82E-005
31.1	chr12	44452706	44643337	14	7.2	2.45E-008	3.96E-004
31.0	chr7	30329660	30373973	8	17.9	2.56E-008	9.95E-004
31.0	chr6	69489375	69532764	8	17.8	2.61E-008	7.95E-004
31.0	chr7	87299930	87382867	10	11.7	2.62E-008	9.81E-004
30.9	chr17	50461350	50504883	8	17.7	2.78E-008	8.89E-005
30.8	chr2	39341670	39534412	14	7.1	2.84E-008	2.13E-003
30.8	chr2	55065127	55481751	20	4.8	2.87E-008	2.02E-003
30.8	chr22	35917376	35977201	8	17.6	2.88E-008	4.82E-005
30.8	chr3	106913333	107048330	12	8.7	2.92E-008	1.57E-003
30.6	chr9	9790634	9951145	13	7.8	3.14E-008	4.16E-004
30.5	chr14	49666196	50203542	23	4.2	3.26E-008	5.24E-004
30.5	chr1	108110523	108332348	15	6.4	3.33E-008	2.25E-003
30.5	chr19	47316584	47322161	4	121.9	3.35E-008	1.05E-004
30.4	chr3	116103531	116167708	9	13.6	3.60E-008	1.84E-003
30.1	chr14	69156842	69244970	10	11.2	4.01E-008	5.95E-004
30.1	chr9	5691763	5992967	17	5.5	4.05E-008	4.68E-004
30.1	chrX	24073768	24105069	7	22.3	4.16E-008	6.15E-004
30.1	chr2	171585272	171695854	11	9.6	4.17E-008	2.79E-003
30.1	chr17	37259004	37310239	8	16.8	4.20E-008	1.26E-004
30.0	chr16	24469307	24478639	5	53.6	4.33E-008	2.14E-004
29.9	chr3	95235584	95303379	9	13.3	4.61E-008	2.27E-003
29.8	chr2	63906770	64044804	12	8.4	4.67E-008	2.98E-003
29.8	chr20	340488	460732	11	9.5	4.88E-008	1.88E-004
29.6	chr7	151496453	151988664	21	4.4	5.19E-008	1.81E-003
29.6	chrX	70153838	70250968	10	10.9	5.36E-008	7.56E-004
29.5	chr2	17728138	17819398	10	10.9	5.46E-008	3.36E-003
29.5	chr5	118551972	118897085	18	5.1	5.65E-008	2.43E-003
29.4	chr12	78694293	78743355	8	16.1	5.77E-008	8.48E-004
29.4	chr14	34328407	34843859	22	4.2	5.94E-008	8.49E-004
29.4	chr20	31584157	31675648	10	10.8	5.97E-008	2.13E-004
29.2	chr4	68084809	68231055	12	8.1	6.63E-008	3.11E-003
29.0	chr1	159080068	159090503	5	48.2	7.38E-008	4.76E-003
28.7	chr2	69918241	70011717	10	10.4	8.39E-008	4.92E-003
28.6	chr7	10987276	11136005	12	7.9	8.87E-008	2.97E-003
28.4	chr14	23759975	23773208	5	45.4	9.95E-008	1.25E-003
28.4	chr18	20999949	21094336	10	10.2	1.01E-007	7.27E-004
28.3	chr3	128981837	128994503	5	45.2	1.02E-007	4.93E-003
28.3	chr6	119542181	119724940	13	7.0	1.05E-007	3.08E-003
28.1	chr1	2153470	2326945	10	10.0	1.17E-007	7.55E-003
28.0	chr1	114073650	114171790	10	10.0	1.20E-007	7.69E-003
28.0	chr7	92720269	92818219	10	10.0	1.22E-007	3.92E-003
27.9	chr7	91454108	91530300	9	11.8	1.26E-007	3.78E-003
27.9	chr7	38182505	38217170	7	18.8	1.31E-007	3.84E-003
27.8	chr15	70564480	70662895	10	9.9	1.32E-007	8.96E-004
27.8	chr1	3753210	3780615	6	26.4	1.38E-007	8.52E-003
27.7	chr2	161889071	161965394	9	11.6	1.44E-007	8.05E-003
27.5	chr14	30438548	30494491	8	14.2	1.54E-007	1.90E-003
27.5	chr8	42937993	43163721	14	6.2	1.54E-007	3.81E-003
27.5	chrX	44679749	44777475	10	9.7	1.55E-007	2.05E-003
27.5	chr8	103286618	103441227	12	7.5	1.56E-007	3.74E-003
27.5	chr4	101008249	101082418	9	11.5	1.61E-007	7.33E-003
27.4	chr4	41708641	41764187	8	14.1	1.63E-007	7.02E-003
27.3	chr19	50824110	51051462	12	7.4	1.73E-007	4.77E-004
27.2	chr6	2668640	2870617	13	6.7	1.82E-007	5.06E-003
27.1	chr13	19171373	19553623	18	4.7	1.96E-007	3.49E-003
27.1	chr7	120778958	120816738	7	17.8	1.97E-007	5.46E-003
26.8	chr16	54035474	54174962	11	8.1	2.22E-007	1.00E-003
26.5	chr20	43044298	43123097	9	10.8	2.63E-007	8.23E-004
26.1	chr17	6453040	6613336	11	7.9	3.16E-007	9.25E-004
26.1	chr14	57745334	58016310	15	5.4	3.19E-007	3.77E-003
26.1	chr18	2534514	2806006	15	5.4	3.26E-007	2.17E-003
25.7	chr16	20721091	20805152	9	10.3	3.93E-007	1.76E-003
25.6	chr11	14679457	14853589	12	6.8	4.12E-007	7.18E-003

25.6	chr17	30427455	30453752	6	21.9	4.22E-007	1.19E-003
25.5	chr8	71429938	71456492	6	21.7	4.51E-007	1.00E-002
25.4	chr14	30108441	30194857	9	10.1	4.64E-007	4.88E-003
25.3	chr9	74956687	74970888	5	33.2	4.87E-007	5.17E-003
25.1	chr11	75264979	75507300	14	5.6	5.46E-007	9.05E-003
24.1	chr21	37370598	37555945	12	6.3	9.34E-007	1.80E-003
23.8	chr22	15681255	15698130	5	28.2	1.09E-006	1.61E-003
23.7	chr15	99554793	99604261	7	13.7	1.14E-006	7.33E-003
23.4	chr19	45012854	45954364	25	3.2	1.32E-006	2.94E-003
23.1	chr20	46830355	47235662	17	4.2	1.57E-006	4.53E-003
22.6	chr20	30429519	30783053	14	5.0	2.01E-006	5.03E-003
22.3	chr19	48945393	48968825	5	24.3	2.32E-006	4.45E-003
22.2	chr22	36582773	36686043	8	9.8	2.50E-006	3.52E-003
21.9	chr22	18418399	18466284	6	15.8	2.92E-006	3.91E-003
