# Supplementary material for: A novel scan statistics approach for clustering identification and comparison in binary genomic data
Source: BMC Bioinformatics. 2016 Sep 22;17(Suppl 11):320. doi: 10.1186/s12859-016-1173-8 (PMC5046198; doi:10.1186/s12859-016-1173-8)
Supplement: Additional file 2 — Table S2. Full list of MLV clusters. (PDF 80 kb) [file 12859_2016_1173_MOESM2_ESM.pdf]

Table 1: List of clusters identified in MLV data by Scan Statistics.

S	Chr	Start	End	IS count	$\frac{p_{M\hat{L}V_Z}}{q_{M\hat{L}V_Z}}$	Raw pvalue	Adj pvalue
386.5	chr20	51646845	51991770	89	22.8	<2E-16	<2E-16
326.4	chr20	10362242	10450134	55	51.8	<2E-16	<2E-16
318.4	chr17	26646082	26672265	41	131.1	<2E-16	<2E-16
302.6	chr17	76325116	76460372	56	39.5	<2E-16	<2E-16
285.6	chr19	59566413	59591310	37	127.9	<2E-16	<2E-16
284.6	chr21	38671040	39311896	90	12.2	<2E-16	<2E-16
279.2	chr17	51718847	53782415	142	6.2	<2E-16	<2E-16
278.7	chr1	25046795	28847012	183	4.7	<2E-16	<2E-16
267.7	chr18	72291047	72971441	87	11.6	<2E-16	<2E-16
264.4	chr12	6084417	10441567	197	4.2	<2E-16	<2E-16
245.8	chr1	232803321	233224771	69	15.1	<2E-16	<2E-16
239.4	chr6	2664481	2808763	49	30.3	<2E-16	<2E-16
228.5	chr11	33859230	33921119	38	54.0	<2E-16	<2E-16
228.2	chr12	117227758	117281237	37	58.3	<2E-16	<2E-16
227.4	chr16	23772880	23871653	43	37.2	<2E-16	<2E-16
227.2	chr14	99590139	99618631	32	93.6	<2E-16	<2E-16
227.2	chr2	43004474	43307705	56	19.6	<2E-16	<2E-16
226.2	chr21	18070783	18088776	29	133.3	<2E-16	<2E-16
206.1	chr17	55215264	55262988	33	60.8	<2E-16	<2E-16
203.9	chr4	81264902	81278530	25	159.3	<2E-16	<2E-16
201.9	chr11	9201752	10722160	105	6.0	<2E-16	<2E-16
201.8	chr18	2948658	3655616	76	9.2	<2E-16	<2E-16
201.6	chr8	108383857	108438367	34	51.7	<2E-16	<2E-16
200.1	chr9	128154606	133603768	180	3.6	<2E-16	<2E-16
199.7	chr19	46742680	46754521	24	173.4	<2E-16	<2E-16
198.5	chr21	14745759	16026968	98	6.4	<2E-16	<2E-16
196.8	chr15	76113355	76148984	30	71.3	<2E-16	<2E-16
195.4	chr3	71515756	71717137	48	19.8	<2E-16	<2E-16
195.2	chr9	78365972	78458984	38	34.5	<2E-16	<2E-16
194.4	chr16	73632186	73668775	30	68.4	<2E-16	<2E-16
190.4	chr17	69970535	72970208	127	4.6	<2E-16	<2E-16
189.4	chr7	2405916	2746552	48	18.5	<2E-16	<2E-16
186.2	chr6	6536008	7259415	72	8.9	<2E-16	<2E-16
186.1	chr16	56128703	56326446	42	23.9	<2E-16	<2E-16
177.3	chr5	427452	459107	25	93.2	<2E-16	<2E-16
176.5	chr1	44653998	44673254	24	106.4	<2E-16	<2E-16
176.4	chr17	24476548	24496317	23	124.9	<2E-16	<2E-16
174.4	chr6	35106484	35125420	24	101.8	<2E-16	<2E-16
174.0	chr6	25105936	26473038	94	5.8	<2E-16	<2E-16
173.5	chr15	72840682	73283286	53	12.9	<2E-16	<2E-16
173.1	chr16	16064630	16092259	25	85.7	<2E-16	<2E-16
172.8	chr3	17913726	18271129	54	12.4	<2E-16	<2E-16
172.7	chr22	35955220	36036598	31	43.1	<2E-16	<2E-16
172.0	chr15	92512419	92650308	39	23.7	<2E-16	<2E-16
170.7	chr19	644536	3140939	94	5.7	<2E-16	<2E-16
169.9	chr1	204403909	204496220	34	32.1	<2E-16	<2E-16
169.6	chr12	11768552	11933620	41	20.5	<2E-16	<2E-16
168.6	chr7	141274116	142818610	95	5.5	<2E-16	<2E-16
168.3	chr2	74651642	74657112	18	290.2	<2E-16	<2E-16
167.4	chr12	120711139	120726945	19	221.7	<2E-16	<2E-16
167.1	chr8	130615573	130762356	39	22.1	<2E-16	<2E-16
166.6	chr16	80221935	80424179	41	19.7	<2E-16	<2E-16
166.2	chr2	85483333	85669721	40	20.7	<2E-16	<2E-16
166.2	chr21	45118620	45172492	25	74.5	<2E-16	<2E-16
166.1	chr19	16050403	16144677	31	38.6	<2E-16	<2E-16
165.4	chr14	70517245	70519252	15	672.4	<2E-16	<2E-16
165.0	chr16	10869713	10893701	23	97.3	<2E-16	<2E-16
162.6	chr22	45366718	45388748	22	108.3	<2E-16	<2E-16
160.2	chr12	51587404	56622291	170	3.2	<2E-16	<2E-16
156.1	chr11	122066684	122103540	25	60.6	<2E-16	<2E-16
155.5	chr1	196846926	197279242	54	10.4	<2E-16	<2E-16
155.1	chr17	6878440	10015076	124	3.9	<2E-16	<2E-16
154.4	chr11	120772616	120966480	40	17.7	<2E-16	<2E-16
154.1	chr19	49936730	50054577	31	31.6	<2E-16	<2E-16
152.4	chr11	64015325	65112168	68	7.3	<2E-16	<2E-16
152.2	chr12	115471635	115609147	35	22.9	<2E-16	<2E-16
151.0	chr4	39732051	40025369	44	14.1	<2E-16	<2E-16
149.8	chr17	25026031	25078706	27	42.5	<2E-16	<2E-16
149.7	chr6	135542359	135687232	36	20.7	<2E-16	<2E-16
149.4	chr13	31513244	31589299	30	31.8	<2E-16	<2E-16
148.7	chr12	74135605	74164204	23	67.9	<2E-16	<2E-16
148.6	chr18	58890820	58957741	28	37.6	<2E-16	<2E-16
148.4	chr20	8066045	8388575	47	12.2	<2E-16	<2E-16
147.8	chr11	34394225	34406511	19	132.0	<2E-16	<2E-16
147.5	chr17	263260	2701238	101	4.5	<2E-16	<2E-16
146.9	chr16	20720244	20835649	33	24.2	<2E-16	<2E-16
145.6	chr14	19961750	24216243	151	3.2	<2E-16	<2E-16
145.5	chr3	87925767	87967828	25	48.9	<2E-16	<2E-16
144.6	chr4	15010539	15668163	61	7.8	<2E-16	<2E-16
144.3	chr1	66569618	66678557	32	24.9	<2E-16	<2E-16
143.7	chr13	76796262	76867522	28	34.4	<2E-16	<2E-16
142.7	chr3	197819979	197853794	22	68.7	<2E-16	<2E-16
142.4	chr9	122874645	123197679	45	12.2	<2E-16	<2E-16
141.8	chr16	65108591	67131656	92	4.8	<2E-16	<2E-16
141.7	chr22	27525356	27930112	48	10.9	<2E-16	<2E-16
141.7	chr5	42985407	43109288	32	23.9	<2E-16	<2E-16

141.4	chr4	10278489	10318151	24	50.7	<2E-16	<2E-16
141.4	chr1	92696128	92754199	26	40.2	<2E-16	<2E-16
140.7	chr16	87275698	88358399	60	7.7	<2E-16	<2E-16
138.3	chr11	117583401	117618858	23	53.9	<2E-16	<2E-16
137.6	chr2	68433915	68851522	49	10.0	<2E-16	<2E-16
137.3	chr17	59809264	59883346	27	33.5	<2E-16	<2E-16
135.8	chr12	554828	731228	35	17.9	<2E-16	<2E-16
134.9	chr9	3496424	3519568	20	78.3	<2E-16	<2E-16
134.6	chr2	45917012	45949914	22	56.9	<2E-16	<2E-16
134.0	chr18	21056370	21185978	32	21.1	<2E-16	<2E-16
133.6	chr10	26764982	26812253	24	43.0	<2E-16	<2E-16
133.6	chr17	4282673	4288254	14	319.6	<2E-16	<2E-16
133.5	chr21	35182496	35340693	34	18.3	<2E-16	<2E-16
133.3	chr15	48133020	48216547	28	28.4	<2E-16	<2E-16
133.2	chr5	75604019	76291404	59	7.3	<2E-16	<2E-16
133.0	chr8	104219745	104278781	25	37.9	<2E-16	<2E-16
132.7	chr6	45500211	45573646	27	30.7	<2E-16	<2E-16
132.5	chr1	153175160	155010062	86	4.8	<2E-16	<2E-16
132.4	chr22	15944311	15978345	21	62.6	<2E-16	<2E-16
132.2	chr12	122788311	122799132	17	131.8	<2E-16	<2E-16
131.1	chr10	21847098	21862242	18	102.8	<2E-16	<2E-16
127.1	chr18	13303768	13619897	41	11.8	<2E-16	<2E-16
126.9	chr10	11196956	12133796	65	6.1	<2E-16	<2E-16
126.0	chrX	153596778	153682348	27	27.0	<2E-16	<2E-16
125.9	chr12	67041204	67062352	19	73.6	<2E-16	<2E-16
125.6	chr18	27467193	27523385	24	36.2	<2E-16	<2E-16
125.6	chr16	68993868	69022290	19	73.0	<2E-16	<2E-16
125.6	chr16	83608178	84577004	58	7.0	<2E-16	<2E-16
124.9	chr4	84248972	84460326	36	14.4	<2E-16	<2E-16
124.7	chr9	20959899	21094304	31	19.3	<2E-16	<2E-16
124.5	chr15	83888742	84051668	33	16.9	<2E-16	<2E-16
124.3	chr11	3759599	3872491	28	24.0	<2E-16	<2E-16
124.2	chr13	90635481	90721238	27	26.1	<2E-16	<2E-16
123.7	chr18	51115731	51174780	24	34.8	<2E-16	<2E-16
123.5	chr1	108130496	108310198	34	15.7	<2E-16	<2E-16
123.1	chr3	4287023	5185795	64	6.0	<2E-16	<2E-16
122.0	chr1	167865661	167947829	26	27.4	<2E-16	<2E-16
121.6	chr20	3715773	5098172	76	4.9	<2E-16	<2E-16
120.8	chr2	70166370	70222433	23	36.5	<2E-16	<2E-16
120.5	chr12	13142244	13251856	28	22.4	<2E-16	<2E-16
119.5	chr4	57672065	57675940	13	268.4	<2E-16	<2E-16
119.5	chr1	206064248	206192299	29	20.3	<2E-16	<2E-16
119.0	chr5	131773768	131857446	25	28.4	<2E-16	<2E-16
118.2	chr4	141093863	141256370	32	16.2	<2E-16	<2E-16
118.1	chr12	14992738	15033661	21	44.3	<2E-16	<2E-16
118.1	chr3	197328368	197405338	24	30.8	<2E-16	<2E-16
117.3	chr16	17343831	17475815	29	19.5	<2E-16	<2E-16
116.6	chr13	27717365	27745278	19	57.5	<2E-16	<2E-16
116.2	chr5	150020458	150151681	29	19.1	<2E-16	<2E-16
115.9	chr16	29561401	31454256	80	4.5	<2E-16	<2E-16
115.9	chr21	42170843	43076414	57	6.4	<2E-16	<2E-16
115.1	chr13	47325932	50389501	118	3.3	<2E-16	<2E-16
114.8	chr10	5315974	7604245	96	3.8	<2E-16	<2E-16
114.7	chr21	37652013	37677369	18	64.8	<2E-16	<2E-16
114.5	chr7	20163407	20308433	30	17.3	<2E-16	<2E-16
114.1	chr19	12638188	13136805	40	10.3	<2E-16	<2E-16
113.8	chr20	29568791	31025652	72	4.9	<2E-16	<2E-16
112.9	chr14	34824905	34854876	19	52.0	<2E-16	<2E-16
112.9	chr19	43171448	43238857	23	30.6	<2E-16	<2E-16
112.1	chr15	91152326	91232031	24	27.1	<2E-16	<2E-16
112.1	chr7	76657311	76820967	31	15.5	<2E-16	<2E-16
112.0	chr12	88073767	88271236	33	13.8	<2E-16	<2E-16
112.0	chr11	72532179	72543496	15	112.7	<2E-16	<2E-16
111.7	chr6	147221267	147277346	22	33.4	<2E-16	<2E-16
111.7	chr11	1822695	1908794	20	43.3	<2E-16	<2E-16
111.5	chr14	73277409	73323061	20	43.2	<2E-16	<2E-16
110.7	chr9	213291	746574	48	7.5	<2E-16	<2E-16
110.0	chr2	16456554	16706579	35	12.1	<2E-16	<2E-16
110.0	chr5	14208828	14266689	22	32.1	<2E-16	<2E-16
109.9	chr19	48949700	48981127	17	67.8	<2E-16	<2E-16
109.8	chr9	79695822	79716317	17	67.6	<2E-16	<2E-16
109.5	chr1	244294479	245064453	55	6.3	<2E-16	<2E-16
109.5	chr10	104348666	104382853	18	55.8	<2E-16	<2E-16
109.3	chr7	105496556	105538337	20	40.7	<2E-16	<2E-16
108.7	chrX	65123158	65150370	18	54.7	<2E-16	<2E-16
108.5	chr2	54639589	54672354	19	46.3	<2E-16	<2E-16
108.2	chr2	37615424	38194492	49	7.1	<2E-16	<2E-16
107.5	chr12	27059106	27063230	12	238.3	<2E-16	<2E-16
107.5	chr9	99721141	99744043	17	63.1	<2E-16	<2E-16
107.4	chr3	128952512	131155464	88	3.9	<2E-16	<2E-16
107.2	chr20	42557385	42731685	30	15.2	<2E-16	<2E-16
106.8	chr5	10474121	10852357	39	9.6	<2E-16	<2E-16
106.4	chr17	43081234	45219769	87	3.9	<2E-16	<2E-16
105.8	chr5	40521236	40544888	17	60.0	<2E-16	<2E-16
105.7	chr4	156778517	156824302	20	37.2	<2E-16	<2E-16
105.6	chr2	201690309	201837046	28	16.9	<2E-16	<2E-16
105.4	chr3	49104194	50644803	65	5.0	<2E-16	<2E-16
105.0	chr2	64717719	65028132	37	10.2	<2E-16	<2E-16
104.7	chr5	138609393	139072125	41	8.7	<2E-16	<2E-16
104.7	chr18	27852312	27877862	17	58.1	<2E-16	<2E-16
104.7	chr7	5425974	5562030	24	23.1	<2E-16	<2E-16

104.6	chr17	76813946	78206170	57	5.7	<2E-16	<2E-16
103.8	chr3	173163106	173796550	50	6.6	<2E-16	<2E-16
102.2	chr5	32743564	32771428	17	53.9	<2E-16	<2E-16
100.7	chr7	50225729	50393041	26	17.8	<2E-16	<2E-16
99.9	chr4	55220632	55505401	35	10.3	<2E-16	<2E-16
99.7	chr15	88999657	89046938	19	36.4	<2E-16	<2E-16
99.6	chr17	73758992	73895637	24	20.6	<2E-16	<2E-16
98.9	chr17	37536626	40661766	99	3.3	<2E-16	<2E-16
97.7	chr11	14521536	14653473	26	16.8	<2E-16	<2E-16
97.5	chr19	38442931	38486323	16	56.2	<2E-16	<2E-16
97.4	chr9	124826195	124849932	16	56.0	<2E-16	<2E-16
97.4	chr12	103515231	103672931	27	15.5	<2E-16	<2E-16
97.3	chr9	74953746	74959972	12	155.7	<2E-16	<2E-16
97.2	chr11	37604470	37610843	12	155.2	<2E-16	<2E-16
96.9	chrX	135654467	135694526	18	39.1	<2E-16	<2E-16
96.7	chr4	37426261	37543894	25	17.8	<2E-16	<2E-16
96.7	chr14	50349154	50466230	25	17.8	<2E-16	<2E-16
96.6	chr8	62761791	62809970	19	33.5	<2E-16	<2E-16
95.5	chr13	107664351	107724026	20	28.6	<2E-16	<2E-16
94.9	chr3	69186133	69226961	18	36.9	<2E-16	<2E-16
94.6	chr12	32602858	32636206	17	43.0	<2E-16	<2E-16
94.6	chr6	36949759	37357105	37	8.7	<2E-16	<2E-16
93.7	chr11	77674582	77964993	34	9.7	<2E-16	<2E-16
93.3	chr6	105208792	105283569	21	24.1	<2E-16	<2E-16
92.9	chr3	184409800	184498862	22	21.4	<2E-16	<2E-16
91.9	chr12	2264063	2343891	21	23.2	<2E-16	<2E-16
91.9	chr6	156367019	156712705	36	8.7	<2E-16	<2E-16
91.6	chr13	45639907	45683214	18	33.6	<2E-16	<2E-16
89.6	chr3	161038641	161063019	15	52.8	<2E-16	<2E-16
89.6	chr19	56318973	57099762	47	6.0	<2E-16	<2E-16
88.6	chr11	113447928	113507654	19	27.0	<2E-16	<2E-16
88.4	chr1	158145742	159550360	65	4.2	<2E-16	<2E-16
88.3	chr3	99963204	99966849	10	223.3	<2E-16	<2E-16
88.2	chr7	92090670	92319551	30	10.8	<2E-16	<2E-16
88.1	chr2	196723417	196835340	23	17.4	<2E-16	<2E-16
88.0	chr4	47902062	47911156	12	105.3	<2E-16	<2E-16
87.9	chr12	115966941	115999280	15	50.0	<2E-16	<2E-16
87.8	chr10	17281154	17300175	14	61.6	<2E-16	<2E-16
87.7	chr19	61515799	61525541	12	104.1	<2E-16	<2E-16
87.4	chr11	71470705	71500443	15	49.0	<2E-16	<2E-16
87.1	chr15	99474927	99602685	23	17.0	<2E-16	<2E-16
87.1	chr10	73158692	73784053	42	6.6	<2E-16	<2E-16
87.1	chr2	181872755	182034178	26	13.5	<2E-16	<2E-16
86.8	chr11	95584037	95624176	17	33.9	<2E-16	<2E-16
86.8	chr14	74467904	75079792	43	6.4	<2E-16	<2E-16
86.6	chr6	82526634	82764544	30	10.5	<2E-16	<2E-16
86.6	chr1	41964916	42015313	17	33.7	<2E-16	<2E-16
86.5	chr20	20638084	20672603	16	39.6	<2E-16	<2E-16
86.5	chr22	48437708	49326498	42	6.5	<2E-16	<2E-16
86.3	chr19	6013667	8541840	80	3.5	<2E-16	<2E-16
86.2	chr7	130247023	130393480	25	14.2	<2E-16	<2E-16
85.9	chr1	8660826	9075648	36	7.9	<2E-16	<2E-16
85.6	chr12	24826164	25134078	33	8.9	<2E-16	<2E-16
85.3	chr2	113101395	113361066	30	10.2	<2E-16	<2E-16
84.9	chr6	139639412	140004298	35	8.1	<2E-16	<2E-16
84.6	chr2	197777435	197881083	22	17.6	<2E-16	<2E-16
84.5	chr2	6937583	7116191	26	12.8	<2E-16	<2E-16
84.4	chr5	39203957	39310392	22	17.5	<2E-16	<2E-16
83.8	chrX	19673009	19812981	24	14.5	<2E-16	<2E-16
83.7	chr11	46296419	47619145	60	4.3	<2E-16	<2E-16
83.6	chr5	171468904	171539657	18	26.7	<2E-16	<2E-16
83.4	chr10	90080573	90137483	18	26.6	<2E-16	<2E-16
83.0	chr3	153065597	153494231	37	7.3	<2E-16	<2E-16
82.6	chr15	53298975	53359120	18	25.9	<2E-16	<2E-16
82.2	chr13	29835956	29866226	15	41.0	<2E-16	<2E-16
82.0	chr22	36259496	38245569	68	3.8	<2E-16	<2E-16
82.0	chr18	9395537	9866405	38	6.9	<2E-16	<2E-16
82.0	chr7	73262984	73340781	18	25.5	<2E-16	<2E-16
81.3	chr11	35013209	35085700	19	22.1	<2E-16	<2E-16
81.1	chr14	82788962	82806692	13	60.5	<2E-16	<2E-16
80.9	chr12	131526782	131858221	27	11.1	<2E-16	<2E-16
80.8	chr3	178341891	178931134	42	6.0	<2E-16	<2E-16
80.8	chr20	57950112	58067402	22	16.0	<2E-16	<2E-16
80.7	chr19	60455386	60880799	30	9.4	<2E-16	<2E-16
80.6	chr4	88047607	88153421	21	17.5	<2E-16	<2E-16
80.5	chr8	27225310	27331923	21	17.4	<2E-16	<2E-16
80.4	chr1	114204252	114291867	20	19.2	<2E-16	<2E-16
79.3	chr1	148387956	149310130	50	4.9	<2E-16	<2E-16
79.1	chrX	12877322	13031674	24	13.1	<2E-16	<2E-16
78.8	chr5	100111004	100268900	24	13.0	<2E-16	<2E-16
78.7	chr18	37805494	37833206	14	44.2	<2E-16	<2E-16
78.3	chr14	88697432	89260908	40	6.2	<2E-16	<2E-16
78.1	chr8	24207228	24298381	20	18.1	<2E-16	<2E-16
77.7	chr19	53443109	53453224	11	92.1	<2E-16	<2E-16
77.7	chr11	36334171	36597098	29	9.3	<2E-16	<2E-16
77.7	chr7	26206243	27172300	52	4.6	<2E-16	<2E-16
77.6	chr1	66969323	66991717	13	52.8	<2E-16	<2E-16
76.9	chr12	29147156	29327344	25	11.6	<2E-16	<2E-16
76.8	chr12	64824407	64826339	8	328.4	<2E-16	<2E-16
76.7	chr10	112611591	112624765	11	87.7	<2E-16	<2E-16
76.7	chr14	87486522	87543383	17	24.9	<2E-16	<2E-16

76.5	chr9	116166659	116761538	40	6.0	<2E-16	<2E-16
76.3	chr9	2006361	2151055	23	13.3	<2E-16	<2E-16
76.2	chr7	44984794	45037073	16	28.4	<2E-16	<2E-16
75.7	chr18	1982844	2080500	20	17.0	<2E-16	<2E-16
75.7	chr13	40474184	40490125	12	62.7	<2E-16	<2E-16
75.0	chr12	100693771	100811306	21	15.2	<2E-16	<2E-16
75.0	chr9	5810603	5842958	14	38.6	<2E-16	<2E-16
74.9	chr8	8226211	8242481	12	60.5	<2E-16	<2E-16
73.7	chr20	46772285	46923104	22	13.5	<2E-16	<2E-16
73.7	chr13	102224494	102250164	13	45.2	<2E-16	<2E-16
73.1	chr8	19504850	19530184	13	44.3	<2E-16	<2E-16
72.8	chr3	15455981	15824372	32	7.4	<2E-16	<2E-16
72.1	chr8	67007911	67084706	18	19.1	<2E-16	<2E-16
71.4	chr7	15222289	15227654	9	142.8	<2E-16	<2E-16
71.4	chr1	166737615	166772883	14	33.8	<2E-16	<2E-16
71.2	chr4	57318539	57478193	22	12.7	<2E-16	<2E-16
71.1	chr12	108916444	110384728	60	3.8	<2E-16	<2E-16
70.6	chr7	104374455	104445366	17	20.7	<2E-16	<2E-16
70.5	chr21	16485079	16512260	13	40.0	<2E-16	<2E-16
70.0	chr11	101825031	101939251	20	14.6	1.11E-16	3.73E-12
69.2	chr18	44554916	44835039	26	9.2	1.11E-16	1.05E-12
68.8	chr9	138547621	138672207	16	22.3	1.11E-16	2.94E-12
68.8	chr14	76445625	76635184	22	11.9	1.11E-16	2.67E-12
68.6	chr2	27879302	28071024	24	10.3	1.11E-16	1.20E-11
68.5	chr7	79572685	79674439	19	15.5	1.11E-16	4.47E-12
68.4	chr2	136458292	136617070	22	11.8	1.11E-16	1.08E-11
68.4	chr2	225502528	225524170	12	46.0	1.11E-16	1.02E-11
68.3	chr3	113521891	113597772	17	19.2	1.11E-16	7.90E-12
68.1	chr3	109291184	109331080	14	29.9	1.11E-16	7.44E-12
67.7	chrX	37497863	37559012	16	21.5	2.22E-16	3.83E-12
67.6	chr10	121474922	121536723	16	21.5	2.22E-16	8.92E-12
67.6	chr6	42848755	42860293	10	78.8	2.22E-16	1.29E-11
67.4	chr12	45124024	45155333	13	35.3	2.22E-16	6.86E-12
67.4	chr17	58853925	59512745	36	5.8	2.22E-16	3.73E-12
67.2	chr12	119152978	119174853	11	56.8	2.22E-16	5.97E-12
67.1	chr4	95478127	95494062	11	56.4	2.22E-16	8.14E-12
67.1	chr6	21698463	22260243	37	5.6	2.22E-16	1.24E-11
67.1	chr20	45380137	45428558	14	28.8	2.22E-16	2.10E-12
66.9	chr20	155836	368155	23	10.6	3.33E-16	2.98E-12
66.9	chr6	130047302	130112299	16	20.9	3.33E-16	1.72E-11
66.8	chr13	35818058	35925258	19	14.7	3.33E-16	3.61E-12
66.7	chr18	18902624	19338602	32	6.6	3.33E-16	2.73E-12
66.6	chr10	120840240	120886600	14	28.3	3.33E-16	1.28E-11
66.4	chr3	101273713	101284359	10	74.1	3.33E-16	2.18E-11
66.4	chr6	89787121	91051706	56	3.8	3.33E-16	1.65E-11
66.3	chr4	74790263	74902255	19	14.5	4.44E-16	1.58E-11
66.1	chr16	11183718	12461799	53	3.9	4.44E-16	5.72E-12
65.9	chr2	74897018	74921757	12	41.3	4.44E-16	3.89E-11
65.8	chr11	74718263	74881377	20	13.1	4.44E-16	1.44E-11
65.6	chr8	133841942	134646838	42	4.8	5.55E-16	1.93E-11
65.5	chr10	98337421	98507979	22	11.0	5.55E-16	2.07E-11
65.4	chr1	12417493	12462766	14	27.1	5.55E-16	5.85E-11
65.4	chr9	125999685	126463992	31	6.7	5.55E-16	1.39E-11
65.3	chr3	56566635	56933469	30	7.0	6.66E-16	4.27E-11
65.3	chr19	48757888	48777090	11	51.8	6.66E-16	7.84E-12
65.2	chr3	46107478	46238825	20	12.8	6.66E-16	3.96E-11
65.0	chr5	176658686	176868194	20	12.8	7.77E-16	4.03E-11
65.0	chr10	133984846	134251779	22	10.9	7.77E-16	2.60E-11
64.9	chr21	33330020	33836008	34	6.0	7.77E-16	3.37E-12
64.9	chr19	43800726	44034262	22	10.8	7.77E-16	8.77E-12
64.9	chr6	126165850	126314661	21	11.7	7.77E-16	3.62E-11
64.7	chr5	88725063	88744468	11	50.4	8.88E-16	4.23E-11
64.6	chr14	49119186	49653518	35	5.7	8.88E-16	2.06E-11
64.5	chr2	144987449	145179845	23	10.0	9.99E-16	8.72E-11
64.5	chr14	67499180	70195712	85	2.8	9.99E-16	2.19E-11
64.2	chr9	6932222	6951157	11	49.3	1.11E-15	2.60E-11
63.8	chr22	21489232	23456407	62	3.4	1.33E-15	1.33E-11
63.6	chr7	47500256	47585383	16	18.8	1.55E-15	5.99E-11
63.5	chr22	41704183	41992294	23	9.8	1.67E-15	1.22E-11
63.4	chr2	32667909	32714700	14	25.1	1.67E-15	1.39E-10
63.4	chr19	17048323	18390519	47	4.2	1.78E-15	1.83E-11
63.2	chrX	30503223	30522980	11	47.1	1.89E-15	3.06E-11
63.2	chr1	86229105	86301510	16	18.6	1.89E-15	1.86E-10
61.9	chr1	157242200	157304447	15	20.4	3.66E-15	3.41E-10
61.8	chr2	238249194	238273638	11	44.0	3.89E-15	3.13E-10
61.7	chr2	8285755	8740629	31	6.3	4.11E-15	3.11E-10
61.6	chr9	20366035	20387830	11	43.7	4.22E-15	9.55E-11
61.5	chr5	77814894	77843135	12	34.2	4.44E-15	1.97E-10
61.4	chr4	185490612	186048707	35	5.4	4.66E-15	1.44E-10
61.1	chr8	59670511	60411675	41	4.6	5.33E-15	1.79E-10
61.0	chr2	33516670	33608573	17	15.3	5.66E-15	4.11E-10
61.0	chr11	44538374	44575435	12	33.5	5.66E-15	1.72E-10
60.9	chr22	28907384	29036861	17	15.3	5.88E-15	3.48E-11
60.5	chr10	104930537	104953994	11	41.6	7.22E-15	2.33E-10
60.4	chr8	48443352	48678696	24	8.5	7.66E-15	2.30E-10
60.3	chr5	96294323	96324566	12	32.5	8.10E-15	3.48E-10
60.1	chr3	127728645	127744255	9	75.5	9.10E-15	5.01E-10
60.0	chr7	80577465	80608070	12	32.1	9.33E-15	3.55E-10
59.9	chr14	38367012	38391207	11	40.4	9.99E-15	1.70E-10
59.6	chr10	75337547	75442998	17	14.7	1.17E-14	3.68E-10
59.5	chr17	17045320	17636854	32	5.8	1.21E-14	1.78E-10

59.3	chrX	45467559	45550831	16	16.3	1.34E-14	2.15E-10
59.3	chr4	143626765	143843968	23	8.8	1.37E-14	3.92E-10
59.2	chr17	34105757	36011965	61	3.2	1.44E-14	1.84E-10
59.1	chr11	85514662	85581985	15	18.5	1.51E-14	4.50E-10
59.0	chr7	38176903	38334031	20	10.8	1.58E-14	5.82E-10
58.9	chr8	56848861	56968939	18	12.9	1.62E-14	4.65E-10
58.8	chr15	38386640	38478654	15	18.3	1.77E-14	5.44E-10
58.7	chr11	109405366	109489364	16	16.0	1.82E-14	5.28E-10
58.2	chr19	47396556	47478363	14	20.7	2.35E-14	1.86E-10
58.1	chr12	46484756	46494016	8	101.4	2.54E-14	6.65E-10
58.0	chr19	53814809	53834829	9	67.2	2.63E-14	1.97E-10
57.8	chr6	216908	244706	11	36.7	2.83E-14	1.27E-09
57.8	chr12	93042443	93206240	20	10.5	2.91E-14	7.11E-10
57.5	chr1	45781670	45789633	8	97.7	3.42E-14	3.17E-09
57.4	chr6	74477895	74495234	10	46.9	3.55E-14	1.59E-09
57.2	chr8	120998490	121165531	20	10.3	3.87E-14	1.07E-09
57.2	chr11	68352199	68367517	9	64.3	3.91E-14	1.07E-09
57.0	chr17	64555763	64601869	13	23.4	4.30E-14	4.29E-10
56.5	chr7	115452313	115579570	18	12.0	5.51E-14	1.79E-09
56.4	chr1	219888435	220202543	26	7.0	6.05E-14	5.51E-09
56.3	chr7	149691562	149763984	14	19.3	6.13E-14	1.85E-09
56.2	chr1	165682886	165885496	21	9.3	6.49E-14	5.56E-09
56.2	chr10	64656074	64852246	21	9.3	6.51E-14	1.89E-09
55.9	chr12	49999585	50010406	8	88.5	7.55E-14	1.59E-09
55.9	chr18	50622875	50671546	13	22.3	7.56E-14	4.80E-10
55.8	chr6	12919869	15529016	80	2.6	7.92E-14	3.44E-09
55.8	chr8	38764420	38919748	19	10.8	8.10E-14	2.09E-09
55.8	chr5	118635525	118782997	19	10.8	8.12E-14	3.38E-09
55.7	chr8	1885249	1942504	13	22.2	8.26E-14	1.96E-09
55.7	chr5	32209336	32340451	18	11.7	8.59E-14	3.49E-09
55.6	chr1	21967736	22289695	23	8.0	8.73E-14	7.38E-09
55.6	chr15	55196378	55216016	10	42.8	8.96E-14	2.47E-09
55.3	chr12	95014683	95319498	25	7.2	1.01E-13	2.03E-09
55.3	chr9	73691476	73785635	16	14.3	1.04E-13	2.27E-09
55.0	chr3	31547689	31556962	8	83.7	1.19E-13	6.42E-09
55.0	chr7	40555994	40605972	13	21.5	1.21E-13	3.42E-09
54.9	chr2	239807871	239850305	12	25.8	1.26E-13	9.10E-09
54.8	chr16	71256073	71746942	31	5.5	1.33E-13	1.44E-09
54.8	chr18	69584197	69597311	9	56.0	1.34E-13	8.24E-10
54.7	chr10	63168537	63520127	27	6.4	1.41E-13	3.87E-09
54.5	chr7	1516242	1575357	11	31.4	1.52E-13	4.12E-09
54.3	chr4	5084064	5091894	8	79.8	1.75E-13	4.76E-09
54.3	chr5	111121984	111186307	14	17.8	1.76E-13	7.07E-09
54.1	chr6	107003354	107104625	16	13.7	1.88E-13	5.78E-09
54.1	chr6	162157692	162178757	10	39.6	1.90E-13	5.45E-09
54.1	chr5	134762052	134806564	12	24.9	1.92E-13	7.65E-09
53.8	chr15	61920714	65201132	88	2.5	2.24E-13	5.67E-09
53.8	chr11	58091172	58106700	9	52.9	2.26E-13	5.90E-09
53.3	chr2	127007086	127129527	17	12.0	2.92E-13	2.09E-08
53.1	chr10	101931882	101934210	6	225.0	3.24E-13	8.56E-09
53.0	chr11	32364767	32872786	31	5.3	3.31E-13	7.59E-09
52.8	chr21	46540744	46867638	24	7.1	3.71E-13	1.13E-09
52.7	chr4	68093504	68126683	11	28.9	3.80E-13	9.46E-09
52.6	chr4	22903067	22946576	12	23.3	4.02E-13	9.62E-09
52.6	chr18	74930944	75716989	35	4.6	4.09E-13	2.41E-09
52.6	chr14	90504923	90952792	28	5.9	4.14E-13	6.83E-09
52.4	chr5	140963055	140979136	9	49.0	4.49E-13	1.74E-08
52.3	chr11	129321898	129381307	13	19.3	4.67E-13	1.02E-08
52.3	chrX	78282687	78325805	12	23.0	4.72E-13	6.48E-09
52.1	chr2	104630506	104640634	8	69.4	5.33E-13	3.65E-08
52.0	chr5	95079314	95205810	17	11.5	5.46E-13	2.06E-08
52.0	chr6	18436393	18509000	14	16.4	5.64E-13	1.55E-08
51.8	chr12	119451509	119461934	8	68.4	6.03E-13	1.15E-08
51.8	chr12	47697842	47737377	10	35.2	6.25E-13	1.17E-08
51.7	chr12	60930764	60990859	13	18.8	6.41E-13	1.13E-08
51.7	chr8	52948322	53076184	17	11.4	6.62E-13	1.50E-08
51.5	chrX	70240629	70249624	7	106.9	7.00E-13	9.21E-09
51.2	chr14	92051826	92287402	20	8.7	8.50E-13	1.23E-08
51.1	chr22	20552513	20627828	13	18.4	8.68E-13	4.48E-09
51.1	chr10	30746968	30859643	16	12.4	8.69E-13	2.20E-08
51.0	chr13	98673636	98783612	16	12.4	9.09E-13	9.17E-09
51.0	chr11	104038711	104083839	12	21.8	9.14E-13	1.80E-08
51.0	chr14	99749663	100254772	27	5.9	9.35E-13	1.25E-08
51.0	chr22	25204143	25400069	19	9.3	9.43E-13	4.68E-09
50.9	chr6	124169625	124363921	20	8.7	9.58E-13	2.44E-08
50.8	chr20	1419423	1454967	11	26.4	9.98E-13	7.89E-09
50.8	chr22	15669771	15686388	9	44.8	1.01E-12	4.68E-09
50.8	chr18	58755730	58772377	9	44.6	1.04E-12	5.85E-09
50.7	chr2	207662526	207813129	18	10.1	1.08E-12	6.91E-08
50.4	chr5	80848555	81184564	25	6.4	1.24E-12	4.52E-08
50.3	chr8	6360591	6612493	22	7.5	1.34E-12	2.97E-08
50.0	chr8	90863247	90865927	6	174.8	1.51E-12	3.27E-08
49.9	chr5	169636971	169722618	14	15.1	1.63E-12	5.54E-08
49.9	chr2	105306854	105782574	29	5.3	1.64E-12	9.99E-08
49.7	chr20	47727260	48595917	37	4.2	1.83E-12	1.30E-08
49.6	chr15	67661507	68710640	42	3.8	1.84E-12	3.19E-08
49.2	chr5	88063772	88221791	18	9.6	2.31E-12	7.58E-08
49.1	chr4	38206688	38710377	30	5.1	2.42E-12	5.53E-08
49.1	chr15	41316866	41347934	10	30.6	2.45E-12	3.63E-08
49.0	chr1	38157331	38250643	14	14.6	2.50E-12	2.00E-07
49.0	chr3	30658480	30664997	7	88.7	2.61E-12	1.28E-07

48.9	chr10	44753022	44816672	12	19.8	2.69E-12	6.16E-08
48.8	chr4	26357201	26499066	17	10.4	2.83E-12	6.25E-08
48.7	chr16	86294696	86565299	20	8.1	2.94E-12	2.68E-08
48.7	chr17	74383158	74425689	10	30.1	2.94E-12	2.57E-08
48.6	chr2	172780788	172966749	19	8.7	3.22E-12	1.90E-07
48.3	chr6	27882952	27970652	14	14.2	3.65E-12	8.90E-08
48.1	chr7	129796482	129901967	15	12.5	4.14E-12	1.12E-07
48.0	chr3	43305865	43312891	7	82.9	4.21E-12	2.01E-07
47.8	chr3	9767156	10210415	26	5.7	4.80E-12	2.16E-07
47.7	chrX	128730260	129134971	25	6.0	4.93E-12	6.25E-08
47.7	chr3	196204380	196461256	20	7.9	4.95E-12	2.08E-07
47.6	chr1	205522687	205551181	10	28.4	5.12E-12	3.69E-07
47.6	chr8	29259698	29711389	28	5.3	5.34E-12	1.12E-07
47.5	chr2	175166036	175207264	11	22.5	5.55E-12	2.99E-07
47.5	chr21	20549296	20553319	6	140.8	5.61E-12	1.49E-08
47.4	chr18	5447635	5504658	12	18.6	5.70E-12	3.05E-08
47.3	chr19	40145689	41112912	36	4.1	6.08E-12	4.33E-08
47.1	chr10	70486702	70769623	22	6.9	6.70E-12	1.48E-07
47.1	chr12	19284956	19326984	11	22.1	6.76E-12	1.10E-07
47.0	chr22	39120884	39190745	12	18.3	6.92E-12	3.18E-08
47.0	chr21	44402572	44451735	10	27.5	7.18E-12	1.85E-08
46.9	chr3	34032788	34074207	11	21.9	7.50E-12	2.99E-07
46.9	chr4	79749328	79941697	19	8.3	7.62E-12	1.62E-07
46.8	chr17	16060041	16133460	13	15.4	7.80E-12	6.61E-08
46.8	chr12	121921991	122085510	16	10.7	7.83E-12	1.18E-07
46.8	chr8	108537999	108580249	11	21.7	8.04E-12	1.50E-07
46.6	chr15	88158164	88165942	7	75.0	8.53E-12	1.10E-07
46.5	chr5	57821573	57876768	12	17.8	9.39E-12	2.96E-07
46.4	chr1	33219121	33339147	15	11.7	9.55E-12	6.86E-07
46.3	chr3	131948556	132058674	15	11.7	9.90E-12	3.67E-07
46.3	chr15	40683478	40698388	8	48.1	1.00E-11	1.26E-07
46.3	chr7	156759621	156890448	15	11.7	1.04E-11	2.71E-07
46.2	chr1	198387692	198478309	14	13.1	1.08E-11	7.28E-07
46.0	chr8	69034689	69048417	8	47.1	1.19E-11	2.20E-07
45.9	chr22	29948594	30073768	15	11.5	1.23E-11	4.79E-08
45.8	chrX	23983398	24077063	14	12.9	1.32E-11	1.44E-07
45.5	chr5	58916834	58990520	13	14.6	1.53E-11	4.38E-07
45.5	chr11	62098314	62380815	20	7.4	1.54E-11	2.94E-07
45.4	chr19	52298617	52352659	11	20.4	1.57E-11	9.60E-08
45.4	chr5	35700861	35709330	7	68.4	1.63E-11	4.50E-07
45.2	chr5	159758931	159833203	13	14.4	1.75E-11	4.49E-07
45.1	chr5	130569543	130629867	12	16.8	1.87E-11	4.33E-07
45.1	chr6	136863945	137148591	22	6.5	1.90E-11	4.46E-07
44.9	chr15	88363410	88380690	8	43.8	2.13E-11	2.55E-07
44.8	chr1	84381680	84540493	17	9.1	2.23E-11	1.46E-06
44.5	chr2	24337562	24431243	14	12.3	2.54E-11	1.35E-06
44.4	chr7	41817109	41894585	13	14.0	2.70E-11	6.84E-07
44.3	chr12	64503598	64641579	16	9.8	2.84E-11	4.10E-07
44.1	chr11	17055153	17268860	19	7.6	3.20E-11	5.29E-07
44.0	chr6	109222863	109945406	34	4.1	3.35E-11	7.30E-07
44.0	chr12	107479723	107616988	15	10.7	3.36E-11	4.50E-07
43.8	chr14	38640759	38807494	17	8.8	3.55E-11	4.22E-07
43.8	chr6	16778193	16840905	12	15.8	3.71E-11	7.10E-07
43.7	chr10	105507204	105668606	16	9.6	3.81E-11	8.00E-07
43.6	chr14	106222612	106326025	14	11.9	4.06E-11	4.74E-07
43.4	chr17	19815226	19918371	14	11.8	4.47E-11	3.57E-07
43.4	chr8	103739333	103946367	18	8.0	4.53E-11	8.01E-07
43.3	chr2	218789616	218907847	14	11.7	4.67E-11	2.37E-06
43.3	chr16	3569523	3628163	11	18.4	4.67E-11	4.08E-07
43.3	chr1	77649966	77922619	21	6.5	4.78E-11	3.07E-06
43.2	chr6	159210123	159402094	18	7.9	5.06E-11	9.40E-07
43.1	chr14	104557314	105434288	27	4.9	5.28E-11	5.75E-07
42.9	chr5	94430663	94449005	8	38.6	5.80E-11	1.27E-06
42.7	chr21	28925844	29611685	33	4.1	6.32E-11	1.51E-07
42.7	chr6	116818340	116904826	13	13.0	6.42E-11	1.15E-06
42.6	chr10	116497580	116551063	11	17.8	6.72E-11	1.37E-06
42.6	chr8	142167911	142497687	19	7.3	6.86E-11	1.15E-06
42.5	chr9	99784143	100058861	20	6.8	7.22E-11	1.49E-06
42.3	chr22	35174775	35255854	12	14.8	7.79E-11	2.59E-07
42.3	chr8	62943188	62981529	10	21.5	7.85E-11	1.17E-06
42.3	chr9	27432391	27710221	21	6.4	7.93E-11	1.61E-06
42.1	chr16	82318050	82428816	14	11.2	8.53E-11	6.25E-07
42.1	chr14	64579173	64635094	11	17.4	8.88E-11	8.15E-07
42.0	chr1	39430844	39501150	12	14.6	8.96E-11	5.61E-06
41.9	chr17	55788153	55830316	10	21.1	9.55E-11	7.02E-07
41.9	chr8	102189965	102221220	9	26.8	9.77E-11	1.44E-06
41.6	chr4	119312843	119422125	14	11.0	1.12E-10	2.22E-06
41.5	chr9	94069231	94096845	9	26.2	1.19E-10	2.28E-06
41.5	chr14	101386235	101414986	9	26.2	1.20E-10	9.90E-07
41.5	chr5	61908570	61914765	6	85.0	1.21E-10	2.58E-06
41.4	chr16	51647048	51801561	16	8.9	1.21E-10	8.33E-07
41.4	chr1	40622249	40634694	7	51.2	1.26E-10	7.84E-06
41.4	chr9	74811263	74816845	6	84.3	1.27E-10	2.09E-06
41.3	chr8	95943721	95987067	10	20.4	1.28E-10	1.80E-06
41.1	chr2	224951045	224980613	9	25.7	1.43E-10	6.81E-06
41.0	chr5	178868178	179430437	27	4.7	1.49E-10	3.03E-06
40.8	chr8	37712558	37801801	12	13.9	1.65E-10	2.12E-06
40.8	chr5	36660259	37147568	27	4.7	1.72E-10	3.24E-06
40.7	chr16	27122405	27246532	14	10.6	1.73E-10	1.03E-06
40.6	chr11	128005376	128100464	13	11.9	1.83E-10	2.76E-06
40.6	chr6	154613480	154796512	17	7.9	1.86E-10	3.03E-06

40.4	chr2	74048869	74118180	11	16.1	2.03E-10	9.59E-06
40.2	chr2	88938449	88951833	7	47.0	2.27E-10	1.07E-05
40.2	chr2	55083020	55197283	14	10.4	2.33E-10	1.08E-05
40.2	chr1	108990420	109204248	18	7.2	2.34E-10	1.44E-05
40.0	chr4	47139581	47159991	8	32.2	2.50E-10	4.58E-06
40.0	chr12	44350452	44410183	11	15.7	2.57E-10	3.31E-06
39.9	chr19	10074729	11570386	41	3.3	2.61E-10	1.43E-06
39.9	chr6	10628842	10743600	14	10.3	2.68E-10	3.99E-06
39.9	chr7	17033152	17240474	18	7.2	2.71E-10	6.77E-06
39.9	chrX	117515265	117651449	15	9.2	2.73E-10	2.83E-06
39.8	chr12	49605601	49613161	6	74.2	2.75E-10	3.37E-06
39.7	chr6	35743742	35809035	11	15.5	3.01E-10	4.31E-06
39.5	chr3	159553707	159556807	5	140.8	3.20E-10	1.16E-05
39.3	chr12	31758894	31793117	9	23.1	3.59E-10	4.22E-06
39.2	chr2	237110636	237126040	7	43.8	3.76E-10	1.66E-05
39.0	chr7	80055095	80117049	11	15.0	4.17E-10	9.80E-06
38.8	chr14	60840053	61209866	23	5.2	4.79E-10	3.68E-06
38.6	chr8	82102561	82223440	14	9.8	5.08E-10	6.32E-06
38.6	chr3	37479843	38181127	30	4.0	5.14E-10	1.83E-05
38.4	chr12	15576965	15956086	23	5.2	5.68E-10	6.43E-06
38.4	chr4	164732169	164753944	8	29.0	5.68E-10	1.01E-05
38.3	chr20	61727983	61756379	7	40.8	6.20E-10	3.46E-06
38.2	chr16	48842001	49337769	24	4.9	6.24E-10	3.14E-06
38.2	chr2	160161503	160185991	8	28.6	6.31E-10	2.74E-05
38.2	chr15	89173706	89272990	11	14.4	6.32E-10	7.52E-06
38.2	chr7	18336424	18344571	6	64.5	6.42E-10	1.50E-05
38.1	chr7	100666823	100672046	5	121.4	6.80E-10	1.56E-05
38.0	chr10	14637436	14742472	13	10.7	7.11E-10	1.37E-05
38.0	chr12	119795053	120585733	31	3.9	7.23E-10	7.81E-06
37.8	chr12	63836261	63962492	14	9.4	7.91E-10	8.15E-06
37.8	chr3	72420671	72576738	15	8.5	7.92E-10	2.67E-05
37.6	chr10	94441290	94618618	16	7.7	8.80E-10	1.59E-05
37.6	chr3	161063019	161386441	21	5.6	8.81E-10	2.94E-05
37.5	chr11	116148465	116394291	18	6.6	9.26E-10	1.31E-05
37.3	chr11	6613947	6724986	13	10.4	1.02E-09	1.40E-05
37.2	chr4	6741418	7037907	19	6.1	1.09E-09	1.86E-05
37.1	chr1	182873663	183104689	18	6.5	1.14E-09	6.86E-05
37.1	chr8	121796650	122213270	24	4.8	1.15E-09	1.36E-05
36.9	chr2	232187992	232266586	10	16.2	1.25E-09	5.01E-05
36.9	chr10	17501103	17589244	12	11.6	1.27E-09	2.01E-05
36.7	chr10	22932968	23000471	11	13.4	1.34E-09	2.08E-05
36.7	chr4	174325127	174415007	12	11.5	1.36E-09	2.22E-05
36.6	chrX	15676513	15784496	13	10.1	1.46E-09	1.34E-05
36.6	chr1	195971201	196010139	9	19.7	1.47E-09	8.18E-05
36.6	chr4	72028639	72081187	10	15.9	1.47E-09	2.20E-05
36.5	chr9	134219746	134248617	8	25.6	1.54E-09	2.53E-05
36.5	chr19	54611940	55123679	21	5.4	1.55E-09	5.86E-06
36.5	chr2	108571595	108645837	11	13.2	1.55E-09	5.86E-05
36.4	chr3	45656809	45711620	10	15.8	1.59E-09	5.22E-05
36.4	chr16	24659384	25038327	22	5.1	1.64E-09	7.02E-06
36.3	chr2	143596254	143622908	8	25.3	1.68E-09	6.25E-05
36.1	chr6	47092940	47121169	8	25.0	1.85E-09	2.57E-05
36.1	chr2	60446719	60638476	16	7.3	1.85E-09	6.82E-05
35.9	chr13	44858499	44899683	9	18.9	2.09E-09	1.85E-05
35.9	chr2	192211920	192252133	9	18.9	2.13E-09	6.93E-05
35.9	chr9	91114820	91340687	16	7.3	2.13E-09	3.42E-05
35.7	chr12	63083766	63139876	10	15.2	2.28E-09	2.16E-05
35.7	chr5	173223686	173279313	10	15.2	2.28E-09	4.07E-05
35.7	chr11	125264501	125855436	26	4.3	2.34E-09	2.94E-05
35.5	chr7	74996178	75109020	12	10.9	2.51E-09	5.25E-05
35.4	chr3	25665543	25683795	7	33.1	2.69E-09	8.76E-05
35.3	chr20	49544346	49748070	16	7.1	2.79E-09	1.48E-05
35.3	chr17	50689791	50717625	8	23.6	2.88E-09	1.96E-05
35.3	chr16	21505695	21563933	10	14.8	2.89E-09	1.19E-05
35.0	chr9	8638797	8648587	6	49.3	3.25E-09	4.69E-05
35.0	chr3	186929388	186947815	7	32.1	3.29E-09	1.04E-04
35.0	chr5	162821427	162864463	9	17.9	3.37E-09	5.83E-05
34.9	chr12	14427017	14438157	6	49.0	3.41E-09	3.08E-05
34.9	chr2	206659826	206734088	11	12.2	3.51E-09	1.08E-04
34.8	chr16	47201891	47213780	6	48.4	3.66E-09	1.46E-05
34.7	chr7	23353098	23496474	14	8.3	3.93E-09	8.08E-05
34.6	chr15	40571023	40590424	7	31.1	4.14E-09	4.78E-05
34.5	chr1	94329466	95027481	30	3.7	4.19E-09	2.30E-04
34.5	chr5	40369844	40446027	11	12.0	4.26E-09	7.12E-05
34.5	chr19	13809270	13838149	7	30.9	4.26E-09	1.43E-05
34.4	chr6	74346584	74352521	5	83.9	4.44E-09	5.86E-05
34.4	chr2	216572231	216795728	17	6.4	4.48E-09	1.36E-04
34.4	chr1	89922932	90144504	17	6.4	4.50E-09	2.29E-04
34.4	chr2	58699038	58839825	14	8.2	4.59E-09	1.33E-04
34.1	chr1	30988284	31007083	6	45.4	5.36E-09	2.64E-04
34.0	chrX	48211898	48979963	28	3.9	5.39E-09	4.57E-05
34.0	chr12	92508780	92514093	5	80.7	5.41E-09	4.84E-05
34.0	chr4	116413064	116474367	10	13.8	5.62E-09	8.03E-05
33.8	chr6	2072339	2117513	9	16.7	6.17E-09	8.03E-05
33.8	chr3	69458597	69502880	9	16.7	6.19E-09	1.76E-04
33.7	chr10	111665177	111837272	15	7.3	6.44E-09	9.31E-05
33.6	chr8	30067477	30079156	6	43.9	6.60E-09	7.06E-05
33.6	chr4	170392285	170453953	10	13.6	6.65E-09	9.06E-05
33.3	chr2	98712796	98746556	8	20.8	7.88E-09	2.24E-04
33.3	chr3	156752563	156901043	14	7.9	7.92E-09	2.23E-04
33.2	chr18	30863712	30884373	7	28.1	8.33E-09	4.26E-05

33.1	chr12	2594008	2791598	15	7.1	8.78E-09	7.32E-05
33.0	chr12	112129715	112165572	8	20.4	9.17E-09	6.90E-05
32.9	chr11	122103540	122439501	20	5.1	9.73E-09	1.04E-04
32.8	chr9	134847522	134857272	5	71.6	9.98E-09	1.40E-04
32.8	chr6	119713239	119715672	4	163.4	1.01E-08	1.31E-04
32.6	chr14	94692654	94725696	8	19.9	1.10E-08	6.72E-05
32.6	chr7	137940561	137946465	5	69.5	1.16E-08	2.27E-04
32.4	chr1	201041264	201204333	14	7.6	1.27E-08	6.16E-04
32.2	chr14	77092685	77143621	9	15.2	1.42E-08	7.99E-05
32.1	chr4	90406397	91041818	28	3.7	1.49E-08	1.91E-04
32.0	chr19	52679702	52683086	4	147.4	1.54E-08	4.71E-05
32.0	chr18	41500445	42040347	25	4.0	1.55E-08	7.49E-05
32.0	chr4	124537210	124561127	7	25.6	1.57E-08	1.86E-04
31.9	chrX	118697371	118775253	10	12.3	1.65E-08	1.16E-04
31.9	chr11	63392231	63394664	4	144.8	1.66E-08	1.68E-04
31.7	chr7	22829322	22865014	8	18.7	1.81E-08	3.21E-04
31.7	chr20	38943134	39113375	14	7.4	1.83E-08	9.35E-05
31.6	chr13	26873929	26925531	9	14.7	1.87E-08	1.52E-04
31.6	chr7	20479377	20549444	10	12.1	1.93E-08	3.37E-04
31.5	chr4	159350499	159357307	5	62.6	1.97E-08	2.25E-04
31.5	chr7	37347102	37690332	20	4.9	1.99E-08	3.46E-04
31.4	chr6	41971567	42121965	13	8.0	2.07E-08	2.59E-04
31.4	chr3	58295329	58497395	15	6.7	2.12E-08	5.77E-04
31.3	chr20	3010947	3034871	6	36.0	2.19E-08	1.04E-04
31.3	chr1	54285997	55482962	37	3.0	2.23E-08	1.05E-03
31.3	chr19	44517376	44617650	10	11.9	2.25E-08	6.84E-05
31.2	chr20	33749607	33824504	10	11.9	2.36E-08	1.07E-04
31.2	chr19	8541840	8642298	10	11.9	2.39E-08	6.29E-05
31.1	chr9	94899701	94922471	6	35.3	2.44E-08	3.37E-04
31.1	chr17	6758507	6878440	12	8.9	2.51E-08	1.68E-04
31.0	chr15	23585344	23659857	10	11.7	2.62E-08	2.96E-04
30.9	chr10	115808731	115822847	6	34.5	2.79E-08	3.92E-04
30.7	chr21	34218860	34316428	11	9.9	3.02E-08	5.35E-05
30.7	chr8	53535005	53542103	5	57.4	3.05E-08	3.21E-04
30.7	chr3	116104975	116112227	5	57.4	3.06E-08	7.98E-04
30.5	chr16	4463664	4506613	8	17.3	3.28E-08	1.24E-04
30.5	chr1	9872674	10056940	14	7.0	3.38E-08	1.46E-03
30.3	chr9	79716317	79937968	16	5.9	3.68E-08	4.87E-04
30.3	chr5	67570912	67712643	13	7.7	3.68E-08	6.12E-04
30.3	chr3	103138479	103178249	8	17.0	3.73E-08	9.17E-04
30.2	chr21	25701714	25867731	14	6.9	3.85E-08	6.29E-05
30.0	chr2	66519384	66661152	13	7.6	4.29E-08	1.16E-03
30.0	chr7	145982196	146038526	9	13.4	4.30E-08	7.04E-04
30.0	chr20	8996532	9093379	11	9.6	4.38E-08	1.58E-04
30.0	chr1	241494067	241725017	16	5.8	4.39E-08	1.86E-03
29.9	chr17	56569415	56577877	5	53.2	4.47E-08	2.81E-04
29.8	chr21	37520133	37560912	8	16.5	4.71E-08	4.65E-05
29.8	chr10	114471196	114708234	16	5.8	4.77E-08	6.55E-04
29.8	chr3	47004120	47034787	6	31.5	4.84E-08	1.15E-03
29.8	chr1	227230293	227245967	6	31.5	4.86E-08	1.89E-03
29.7	chr2	225577988	225594397	6	31.3	5.02E-08	1.34E-03
29.7	chr10	104181719	104200986	6	31.1	5.17E-08	6.98E-04
29.6	chr5	33250043	33277721	7	21.5	5.38E-08	8.27E-04
29.5	chr1	190748858	190789827	8	16.1	5.62E-08	1.96E-03
29.5	chrX	152841586	152938953	9	12.9	5.69E-08	3.73E-04
29.4	chr4	84595410	84771113	14	6.7	5.88E-08	6.52E-04
29.4	chr2	62210011	62448020	16	5.7	6.02E-08	1.60E-03
29.4	chr7	138370615	139208443	30	3.3	6.04E-08	9.72E-04
29.3	chr14	59021539	59120111	11	9.2	6.26E-08	3.37E-04
29.1	chr19	46460994	46627106	12	8.1	6.71E-08	1.71E-04
29.0	chr17	30723706	31153054	21	4.3	7.30E-08	4.34E-04
28.9	chr2	128949204	129192370	15	6.0	7.44E-08	1.89E-03
28.9	chr12	88640097	88719566	10	10.5	7.51E-08	5.34E-04
28.9	chr11	66776543	67210941	17	5.3	7.78E-08	7.82E-04
28.8	chr13	32999612	33016193	6	28.9	8.14E-08	5.79E-04
28.7	chr2	31969051	31972639	4	97.5	8.36E-08	2.03E-03
28.6	chr2	187458665	187462203	4	96.5	8.71E-08	2.09E-03
28.6	chr6	18015469	18059110	8	15.2	8.81E-08	1.04E-03
28.6	chr19	45912542	45949390	7	19.9	8.95E-08	2.06E-04
28.6	chr8	144343236	144508344	11	8.9	8.96E-08	9.32E-04
28.6	chr3	5446388	5488944	8	15.2	9.10E-08	2.13E-03
28.6	chr15	56556341	56601278	8	15.2	9.11E-08	1.02E-03
28.5	chr5	177575889	177602230	6	28.1	9.53E-08	1.44E-03
28.4	chr5	56059712	56078108	6	28.1	9.65E-08	1.45E-03
28.4	chr5	164546436	164699087	13	7.0	9.74E-08	1.42E-03
28.4	chr10	42945404	42976027	7	19.7	9.80E-08	1.31E-03
28.4	chr17	4288254	4341997	7	19.6	9.86E-08	4.92E-04
28.4	chr7	116294704	116324820	7	19.6	9.93E-08	1.53E-03
28.4	chr3	142684418	142703203	6	27.9	9.99E-08	2.33E-03
28.4	chr2	233633649	233969034	17	5.2	1.00E-07	2.35E-03
28.3	chrX	9269918	9280509	5	45.1	1.03E-07	6.41E-04
28.3	chr7	8154826	8184496	7	19.5	1.03E-07	1.57E-03
28.3	chr10	23779015	23884453	11	8.8	1.06E-07	1.40E-03
28.3	chr10	1023537	1091213	9	12.0	1.06E-07	1.38E-03
28.2	chr11	70836216	70840913	4	91.4	1.09E-07	1.01E-03
28.2	chr2	191594062	191638611	8	14.8	1.09E-07	2.53E-03
28.1	chr4	11080270	11737555	27	3.4	1.16E-07	1.28E-03
28.0	chr1	15341656	17638689	51	2.3	1.22E-07	4.17E-03
28.0	chr8	8902067	8986546	10	9.9	1.24E-07	1.22E-03
27.9	chr1	98071404	98158619	10	9.9	1.29E-07	3.96E-03
27.9	chr9	137955151	138000234	7	18.9	1.31E-07	1.68E-03

27.7	chr11	5662040	5795982	12	7.6	1.39E-07	1.27E-03
27.7	chr10	76289252	76293459	4	85.7	1.42E-07	1.77E-03
27.7	chr9	119520599	119585898	9	11.6	1.42E-07	1.79E-03
27.7	chr1	13900298	13932361	7	18.6	1.43E-07	4.23E-03
27.6	chr5	149773529	149848172	9	11.6	1.45E-07	2.07E-03
27.6	chr13	20525947	20547639	6	26.1	1.48E-07	1.04E-03
27.5	chr1	35334747	36721422	40	2.6	1.54E-07	4.56E-03
27.5	chr15	79079572	79380595	17	5.0	1.55E-07	1.57E-03
27.5	chr11	71429214	71432928	4	83.8	1.55E-07	1.37E-03
27.5	chr9	70649651	70841096	14	6.2	1.56E-07	1.91E-03
27.5	chr1	100583878	100836268	16	5.3	1.61E-07	4.52E-03
27.2	chr10	99070283	99175478	10	9.5	1.83E-07	2.11E-03
27.2	chr5	148107382	148439608	18	4.7	1.86E-07	2.56E-03
27.2	chrX	39844040	39922227	9	11.2	1.87E-07	1.11E-03
27.2	chr15	52760018	52897307	12	7.4	1.88E-07	1.66E-03
27.1	chr17	3805667	3834422	6	25.1	1.89E-07	9.10E-04
27.1	chr7	99857931	99872945	5	39.8	1.94E-07	2.75E-03
27.1	chr2	48307319	48534685	15	5.6	1.97E-07	4.50E-03
27.0	chr3	51983366	52296472	15	5.6	2.00E-07	4.27E-03
27.0	chr17	23524713	23544377	6	24.7	2.08E-07	9.76E-04
26.9	chr17	60071679	60140772	9	11.1	2.09E-07	8.69E-04
26.9	chr1	37712255	37753342	7	17.6	2.10E-07	5.77E-03
26.9	chr22	31354011	31450795	10	9.4	2.19E-07	5.73E-04
26.8	chr6	135774755	135785652	5	38.8	2.22E-07	2.60E-03
26.8	chr12	12741942	12832749	10	9.3	2.29E-07	1.55E-03
26.7	chr16	77665421	77754903	10	9.3	2.33E-07	8.39E-04
26.7	chr6	54930951	55021813	10	9.3	2.39E-07	2.79E-03
26.6	chr1	181706510	181825049	11	8.0	2.49E-07	6.81E-03
26.6	chr10	119091388	119163291	9	10.9	2.51E-07	2.83E-03
26.4	chr9	33956979	34168253	14	5.9	2.75E-07	3.28E-03
26.3	chr7	81238274	81353958	11	7.9	2.93E-07	3.95E-03
26.3	chr5	156460706	156632236	13	6.4	2.98E-07	3.68E-03
26.2	chr14	51381872	51525660	12	7.0	3.04E-07	1.45E-03
26.2	chrX	77468480	77797326	18	4.5	3.10E-07	1.59E-03
26.1	chr3	48524012	48648547	9	10.5	3.28E-07	6.82E-03
26.0	chr11	93092011	93114159	6	22.6	3.47E-07	3.05E-03
25.9	chr6	149418531	149429951	5	35.4	3.51E-07	3.82E-03
25.8	chr7	101170413	101891470	25	3.4	3.74E-07	4.89E-03
25.8	chr6	36499866	36623834	11	7.7	3.77E-07	3.90E-03
25.8	chr11	94440945	94747259	17	4.7	3.77E-07	3.03E-03
25.7	chr2	196055447	196060265	4	66.8	3.92E-07	8.52E-03
25.7	chr9	115152247	115319178	12	6.8	4.08E-07	4.01E-03
25.5	chr5	17452245	17490502	7	15.8	4.34E-07	4.90E-03
25.5	chr7	149991541	150014165	6	21.7	4.46E-07	5.58E-03
25.4	chr13	71272464	71351202	9	10.1	4.60E-07	2.91E-03
25.4	chr15	97205102	97870710	25	3.4	4.75E-07	3.96E-03
25.3	chr12	40605787	40611213	4	63.3	4.87E-07	3.21E-03
25.3	chr7	106290461	106471838	13	6.1	4.89E-07	5.99E-03
25.2	chr15	50049737	50341754	16	4.9	5.26E-07	3.87E-03
25.2	chr6	119990620	120111848	11	7.5	5.30E-07	5.41E-03
25.1	chr11	93846754	93970475	11	7.4	5.48E-07	4.14E-03
25.0	chr19	47073800	47082425	4	60.9	5.73E-07	1.29E-03
25.0	chr22	42721000	42909435	12	6.6	5.83E-07	1.35E-03
24.8	chr11	73150167	73177606	6	20.5	6.25E-07	4.67E-03
24.8	chr17	62672313	62907102	14	5.5	6.39E-07	2.47E-03
24.8	chr6	34733601	34772897	7	14.9	6.48E-07	6.52E-03
24.6	chr5	66513964	66528268	5	30.9	6.91E-07	7.08E-03
24.6	chr10	22580508	22663374	9	9.6	6.93E-07	7.74E-03
24.6	chr22	44003971	44110759	9	9.6	7.00E-07	1.37E-03
24.6	chr15	28985264	29528669	21	3.7	7.01E-07	4.57E-03
24.4	chr8	61868667	62089110	14	5.4	8.01E-07	7.16E-03
24.3	chr16	1411276	2258334	21	3.7	8.14E-07	2.62E-03
24.3	chr7	112545478	112570544	6	19.6	8.23E-07	9.38E-03
24.2	chr7	48399339	48480766	9	9.4	8.71E-07	9.46E-03
24.1	chr9	20476028	20502603	6	19.2	9.26E-07	8.37E-03
23.9	chr17	41612819	41628267	5	28.8	9.93E-07	3.37E-03
23.6	chr19	13956810	13976338	5	27.8	1.18E-06	2.56E-03
23.4	chr12	107739491	107756160	5	27.2	1.33E-06	8.42E-03
23.4	chr15	32354712	32416282	8	10.7	1.34E-06	8.10E-03
23.3	chr20	15968458	16012930	7	13.3	1.37E-06	4.73E-03
23.1	chr17	22825338	22898705	8	10.5	1.51E-06	4.92E-03
22.9	chr15	73915771	74027454	10	7.5	1.67E-06	9.79E-03
22.9	chr15	77879073	78085416	13	5.5	1.69E-06	9.39E-03
22.4	chr22	45460400	45549512	8	10.0	2.17E-06	3.55E-03
22.3	chr20	24218986	24267879	7	12.4	2.28E-06	7.63E-03
22.2	chr16	28051515	28412953	16	4.3	2.51E-06	6.02E-03
22.1	chr19	14329109	14410598	7	12.1	2.60E-06	5.30E-03
21.6	chr22	18254941	18447279	10	6.9	3.29E-06	5.24E-03
19.3	chr21	26221011	26870243	23	2.9	1.14E-05	9.11E-03
11.8	chrY	2769098	2953953	9	4.1	5.81E-04	3.49E-03
