# Supplementary material for: A novel scan statistics approach for clustering identification and comparison in binary genomic data
Source: BMC Bioinformatics. 2016 Sep 22;17(Suppl 11):320. doi: 10.1186/s12859-016-1173-8 (PMC5046198; doi:10.1186/s12859-016-1173-8)
Supplement: Additional file 3 — Table S3. Full list of relative clusters. (PDF 51 kb) [file 12859_2016_1173_MOESM3_ESM.pdf]

Table 1: List of relative clusters identified by Relative Scan Statistics.

S	Chr	Start	End	HIV IS	MLV IS	$\log(\frac{p_{H\hat{I}V_Z}/q_{H\hat{I}V_Z}}{p_{M\hat{L}V_Z}/q_{M\hat{L}V_Z}})$	Type	Adj pvalue
474.1	chr11	63153734	68347426	659	129	1.91	hiv	<2E-16
450.9	chr6	30095760	33488528	332	7	4.49	hiv	<2E-16
434.2	chr16	95090	3561021	430	41	2.74	hiv	<2E-16
260.9	chr17	70835415	73732441	372	75	1.86	hiv	<2E-16
227.0	chr3	47041751	52978572	422	119	1.47	hiv	<2E-16
219.4	chr9	134493480	139818935	307	60	1.89	hiv	<2E-16
213.5	chr17	77047796	77746204	172	7	3.70	hiv	<2E-16
191.9	chr8	144548769	146194757	182	15	2.89	hiv	<2E-16
122.0	chr19	1027304	6006371	292	104	1.20	hiv	<2E-16
115.4	chr22	48983597	49573459	115	11	2.71	hiv	<2E-16
105.6	chr21	37559632	39311896	9	126	-3.02	mlv	<2E-16
102.1	chr19	54074745	55048471	122	18	2.21	hiv	<2E-16
99.3	chr17	1069411	4213267	229	79	1.23	hiv	<2E-16
96.4	chr1	153550587	154168170	90	7	2.94	hiv	<2E-16
91.8	chr18	70832211	73059134	6	103	-3.26	mlv	<2E-16
91.5	chr17	4573721	7723628	194	62	1.32	hiv	<2E-16
86.5	chr20	49745347	52129713	7	102	-3.07	mlv	<2E-16
86.0	chr12	11729500	14430150	8	105	-2.95	mlv	<2E-16
83.3	chr20	60901158	62379063	109	19	2.02	hiv	<2E-16
81.3	chr6	6536008	13289623	22	141	-2.13	mlv	<2E-16
79.2	chr10	1969742	7267562	19	131	-2.21	mlv	<2E-16
76.6	chr19	9777335	14731512	250	111	0.94	hiv	<2E-16
74.9	chr17	51727727	53335279	14	113	-2.39	mlv	<2E-16
72.5	chr12	115196293	118588671	12	105	-2.49	mlv	<2E-16
72.3	chr1	15530	1800605	87	13	2.19	hiv	<2E-16
71.6	chr1	229815412	233358282	13	107	-2.42	mlv	<2E-16
71.1	chr4	8243456	15668163	29	147	-1.86	mlv	<2E-16
67.7	chr12	46982192	49250083	122	33	1.51	hiv	1.16E-10
66.1	chr4	125895	3323181	108	26	1.64	hiv	1.23E-10
65.3	chr21	14745759	16026968	12	98	-2.41	mlv	2.64E-11
63.2	chr9	129882715	130930659	86	16	1.94	hiv	5.66E-10
63.1	chr10	11086410	14919539	15	104	-2.22	mlv	3.89E-10
62.5	chr5	103738213	110871321	70	9	2.36	hiv	9.02E-10
62.0	chr18	3003641	5466739	9	86	-2.59	mlv	2.47E-10
61.6	chr2	68332043	69243703	1	56	-4.62	mlv	2.83E-09
60.7	chr16	28475205	30674055	135	45	1.27	hiv	1.63E-09
60.5	chr17	39819761	40110816	45	1	4.38	hiv	2.47E-09
59.5	chr11	33850576	36581475	26	127	-1.82	mlv	4.21E-09
59.2	chr11	72207695	72525295	48	2	3.66	hiv	4.27E-09
59.1	chr21	41407031	43268642	3	63	-3.49	mlv	4.49E-10
58.8	chr16	71444662	73951194	9	83	-2.55	mlv	3.61E-09
57.8	chr1	204420604	206166849	10	85	-2.45	mlv	2.83E-08
56.1	chrX	150319347	153589032	76	14	1.95	hiv	7.04E-09
55.8	chr3	69210958	72537443	23	116	-1.86	mlv	3.56E-08
55.8	chr9	248547	2016218	2	56	-3.82	mlv	1.78E-08
55.3	chr20	3940258	5049318	5	67	-2.98	mlv	8.02E-09
54.9	chr16	80070773	82677762	6	70	-2.82	mlv	2.53E-08
54.6	chr16	86571318	88472609	154	62	1.05	hiv	2.74E-08
53.6	chr6	135428999	140203026	38	146	-1.54	mlv	8.43E-08
53.1	chr5	176293705	176632415	47	3	3.17	hiv	1.06E-07
52.2	chr2	42959782	43307705	3	57	-3.38	mlv	3.13E-07
52.0	chr20	8066045	10459698	29	125	-1.68	mlv	3.68E-08
51.9	chr22	27525356	27930112	1	48	-4.44	mlv	4.78E-08
51.6	chr16	17948123	19035576	39	1	4.22	hiv	9.90E-08
51.1	chr22	38787587	40407394	94	26	1.48	hiv	6.54E-08
50.8	chr15	38697407	41252418	129	48	1.14	hiv	2.02E-07
49.5	chr19	61576209	63738924	62	10	2.10	hiv	5.36E-07
49.0	chr17	73900078	76320422	92	26	1.46	hiv	7.96E-07
48.7	chr5	150120313	154114504	2	50	-3.70	mlv	8.75E-07
48.5	chr13	45529113	49164785	31	125	-1.60	mlv	3.98E-07
48.2	chr17	25480269	26637802	61	10	2.08	hiv	1.10E-06
48.0	chr16	14080288	15883219	63	11	2.01	hiv	5.61E-07
47.4	chr17	53783727	54700878	43	3	3.07	hiv	1.40E-06
47.2	chr17	37619828	37680552	36	1	4.13	hiv	1.45E-06
46.8	chr1	242127473	245779333	20	99	-1.83	mlv	7.28E-06
46.7	chr4	37426261	41633764	33	127	-1.55	mlv	2.11E-06
46.5	chr14	99590139	100254772	5	59	-2.83	mlv	1.67E-06
46.4	chr3	178341891	179138187	2	48	-3.65	mlv	4.15E-06
46.0	chr17	23416076	24299709	75	18	1.64	hiv	2.38E-06
45.5	chr5	36276059	43109288	47	153	-1.35	mlv	4.44E-06
44.9	chr13	67029008	71079440	44	4	2.76	hiv	1.81E-06
44.6	chr10	76639196	80753532	4	54	-2.99	mlv	4.22E-06
44.6	chr1	190802755	195897463	58	10	2.02	hiv	2.28E-05
44.2	chr4	84248972	84771113	3	50	-3.23	mlv	6.95E-06
44.1	chr8	103667547	104476340	3	50	-3.23	mlv	5.78E-06
43.6	chr13	27043279	31589299	31	119	-1.54	mlv	3.24E-06
43.1	chr9	116119828	119531358	6	59	-2.62	mlv	1.14E-05
43.0	chr2	37615424	38194492	3	49	-3.21	mlv	3.14E-05
42.8	chr7	140318592	142818610	23	101	-1.70	mlv	2.13E-05
42.4	chr11	61661337	62398734	77	21	1.50	hiv	2.00E-05
42.2	chr8	134062018	135000134	1	40	-4.24	mlv	1.42E-05
42.1	chr4	149579279	152091900	42	4	2.71	hiv	1.73E-05
42.1	chr2	182447419	188061707	62	13	1.80	hiv	4.91E-05

42.0	chr2	84975195	85936490	7	61	-2.49	mlv	4.78E-05
41.7	chr11	13440604	14614211	2	44	-3.55	mlv	2.65E-05
41.6	chr4	54623393	57675940	20	93	-1.76	mlv	2.17E-05
41.3	chrX	78415246	83616451	32	1	3.99	hiv	1.16E-05
41.1	chr5	74939823	76291404	8	63	-2.37	mlv	3.54E-05
41.1	chr12	102799285	107616988	13	76	-2.03	mlv	6.69E-05
41.1	chr17	26659383	26681363	1	39	-4.21	mlv	2.69E-05
41.0	chr2	16362838	16706579	1	39	-4.21	mlv	7.83E-05
40.9	chr17	8780184	10084867	6	57	-2.58	mlv	2.81E-05
40.8	chr3	78410138	86370908	41	4	2.68	hiv	6.70E-05
40.6	chr18	13127913	13619897	2	43	-3.52	mlv	1.11E-05
40.5	chr2	113017032	114168629	2	43	-3.52	mlv	9.64E-05
40.1	chr10	128806723	134215283	8	62	-2.35	mlv	3.99E-05
40.1	chr12	6375388	7556157	102	38	1.14	hiv	1.07E-04
39.8	chr12	131713150	131837603	31	1	3.95	hiv	9.94E-05
39.8	chr12	121539154	121821073	31	1	3.95	hiv	9.86E-05
39.8	chr3	187419550	191821498	10	67	-2.18	mlv	1.16E-04
39.7	chr17	31996632	33855903	54	10	1.94	hiv	4.93E-05
39.7	chr15	89569474	92650308	21	93	-1.71	mlv	4.74E-05
39.7	chr6	96145275	101394434	56	11	1.87	hiv	9.32E-05
39.6	chr14	67714840	69152895	3	46	-3.13	mlv	5.19E-05
39.4	chr15	67661507	68710640	2	42	-3.50	mlv	4.82E-05
39.1	chr17	58437063	58838135	34	2	3.26	hiv	6.21E-05
38.6	chr12	109506023	110384728	1	37	-4.15	mlv	1.70E-04
38.6	chr18	74495024	76008391	1	37	-4.15	mlv	2.83E-05
37.8	chr1	42939846	44065678	46	7	2.17	hiv	6.88E-04
37.8	chr8	130615573	131282177	5	51	-2.67	mlv	1.30E-04
37.8	chr16	83608178	85908785	10	65	-2.15	mlv	9.40E-05
37.7	chr20	32445317	33134355	33	2	3.23	hiv	4.30E-05
37.4	chr8	108333639	108438367	1	36	-4.12	mlv	1.51E-04
37.4	chr3	15805394	16906161	1	36	-4.12	mlv	3.61E-04
37.4	chr13	86332487	90578383	56	12	1.77	hiv	5.73E-05
37.4	chr16	65272957	68825313	158	81	0.77	hiv	1.10E-04
37.1	chr22	35993901	36430074	2	40	-3.44	mlv	6.35E-05
36.9	chr22	28376309	28891106	29	1	3.88	hiv	5.66E-05
36.9	chr11	120772616	125855436	45	137	-1.28	mlv	2.90E-04
36.8	chr9	76946859	78458984	6	53	-2.50	mlv	2.70E-04
36.8	chr21	34669166	36476044	5	50	-2.64	mlv	3.50E-05
36.5	chr14	74467904	77143621	20	87	-1.69	mlv	2.40E-04
36.3	chr17	24607215	24881542	32	2	3.19	hiv	2.37E-04
36.3	chr19	41183038	42523965	32	2	3.19	hiv	4.20E-04
36.2	chr7	2614008	2753878	1	35	-4.08	mlv	5.60E-04
36.2	chr16	56197361	56707394	1	35	-4.08	mlv	1.11E-04
35.9	chr17	68879524	70197892	2	39	-3.41	mlv	2.76E-04
35.8	chr1	157242200	159550360	19	84	-1.71	mlv	1.83E-03
35.5	chr1	163292392	170710074	37	120	-1.35	mlv	2.06E-03
35.4	chr16	3634119	3861615	28	1	3.83	hiv	1.50E-04
35.4	chr3	88283671	95257809	28	1	3.83	hiv	9.22E-04
35.4	chr19	59768142	60453081	28	1	3.83	hiv	5.95E-04
35.3	chr1	89510356	95027481	35	116	-1.37	mlv	2.11E-03
35.3	chr2	233633649	239812504	17	79	-1.76	mlv	1.38E-03
35.3	chr5	112083920	118601535	56	13	1.68	hiv	6.62E-04
35.1	chr15	47391175	48339343	3	42	-3.03	mlv	4.01E-04
35.1	chr11	111248801	116270578	8	57	-2.25	mlv	6.02E-04
35.0	chr5	138831850	139637124	1	34	-4.05	mlv	7.21E-04
34.9	chr3	172629552	173952676	10	62	-2.09	mlv	1.18E-03
34.9	chr2	3502740	12613615	39	123	-1.32	mlv	1.61E-03
34.9	chr4	166367772	169636682	31	2	3.15	hiv	6.23E-04
34.8	chr15	76113355	76516612	2	38	-3.38	mlv	4.59E-04
34.7	chr9	99734516	101715602	6	51	-2.46	mlv	7.22E-04
34.6	chr10	22461465	26812253	16	76	-1.79	mlv	6.50E-04
34.1	chr6	25105936	25561675	8	56	-2.23	mlv	1.57E-03
34.0	chr10	70486702	73784053	20	84	-1.65	mlv	8.31E-04
34.0	chr19	8203796	8450493	27	1	3.79	hiv	1.12E-03
34.0	chr20	2645483	2927673	27	1	3.79	hiv	2.52E-04
33.9	chr2	26454993	28379427	105	45	0.98	hiv	2.61E-03
33.9	chr16	23772880	27303429	21	86	-1.62	mlv	3.20E-04
33.8	chr1	25046795	25254658	1	33	-4.02	mlv	4.24E-03
33.8	chr9	21013882	21325912	1	33	-4.02	mlv	1.07E-03
33.8	chr7	20071897	20417688	1	33	-4.02	mlv	1.79E-03
33.8	chr9	125731002	126463992	1	33	-4.02	mlv	1.03E-03
32.8	chr18	9395537	11970227	13	67	-1.88	mlv	5.57E-04
32.6	chr19	49886989	50054577	1	32	-3.98	mlv	2.08E-03
32.5	chr6	13812042	20528522	36	114	-1.32	mlv	3.27E-03
32.5	chr11	71207956	71420783	26	1	3.75	hiv	2.17E-03
32.5	chr18	50644396	51185416	2	36	-3.32	mlv	5.76E-04
32.5	chr1	12455149	15714814	2	36	-3.32	mlv	7.42E-03
32.5	chr8	27225310	28284342	2	36	-3.32	mlv	1.86E-03
32.3	chr7	85316787	87767678	37	5	2.30	hiv	3.81E-03
32.2	chr11	3759599	8980370	22	86	-1.56	mlv	2.51E-03
32.1	chr13	59135561	63602291	29	2	3.08	hiv	7.78E-04
31.9	chr1	44971763	52768738	104	46	0.94	hiv	9.09E-03
31.8	chr18	58755730	58957741	3	39	-2.95	mlv	7.28E-04
31.8	chr5	118731000	122969714	51	12	1.67	hiv	3.57E-03
31.6	chr12	54930936	55367151	47	10	1.78	hiv	6.15E-03
31.5	chr11	54896327	58049894	1	31	-3.94	mlv	3.09E-03
31.4	chr2	74653495	74916028	1	31	-3.94	mlv	7.78E-03
31.4	chr22	25204143	26520008	1	31	-3.94	mlv	8.55E-04
31.3	chr19	59566413	59590705	2	35	-3.29	mlv	3.39E-03
31.3	chr21	33226920	33836008	2	35	-3.29	mlv	4.75E-04
31.3	chr3	4287023	5172079	12	63	-1.90	mlv	7.11E-03

31.2	chr11	208639	1455693	61	18	1.41	hiv	3.52E-03
31.1	chr17	15819790	16047860	25	1	3.70	hiv	3.25E-03
31.1	chr4	106301155	107672950	25	1	3.70	hiv	4.30E-03
31.0	chr4	130079224	137241633	36	5	2.27	hiv	4.26E-03
30.9	chr14	50302188	51849702	7	50	-2.26	mlv	3.85E-03
30.9	chr3	30621736	32449730	7	50	-2.26	mlv	8.56E-03
30.8	chr1	19327528	21959221	72	25	1.22	hiv	1.50E-02
30.8	chr2	196678910	201837046	31	102	-1.37	mlv	1.09E-02
30.7	chr14	88549821	89238305	3	38	-2.92	mlv	3.82E-03
30.4	chr6	153344714	160067515	36	111	-1.29	mlv	8.76E-03
30.3	chr13	90578383	90721238	1	30	-3.91	mlv	1.95E-03
30.3	chr3	56688109	56990507	1	30	-3.91	mlv	1.09E-02
30.3	chr12	369215	706632	1	30	-3.91	mlv	1.03E-02
30.3	chr12	92657080	93206240	1	30	-3.91	mlv	9.94E-03
30.3	chr3	37479843	38181127	1	30	-3.91	mlv	1.07E-02
29.7	chr9	122671601	124638818	15	68	-1.74	mlv	7.09E-03
29.6	chr8	142167911	144434316	3	37	-2.89	mlv	7.35E-03
29.6	chr19	14803007	15385295	24	1	3.66	hiv	7.85E-03
29.6	chr9	90128281	92996221	6	46	-2.34	mlv	6.86E-03
29.4	chr18	18902624	19876684	5	43	-2.47	mlv	2.24E-03
29.4	chr12	122791223	127881095	4	40	-2.64	mlv	1.48E-02
29.3	chr5	32209336	34059548	10	56	-1.98	mlv	1.24E-02
29.2	chr19	269821	987739	54	15	1.47	hiv	9.12E-03
29.1	chr21	17896075	18086581	1	29	-3.87	mlv	1.36E-03
29.1	chr11	74664297	75263476	2	33	-3.22	mlv	9.91E-03
28.9	chr12	52066298	52295533	32	4	2.39	hiv	1.89E-02
28.9	chr16	10787720	11627551	7	48	-2.21	mlv	3.19E-03
28.7	chr15	49941441	53578515	16	69	-1.68	mlv	9.37E-03
28.6	chr20	22850066	25337305	6	45	-2.31	mlv	3.54E-03
28.5	chr2	70150454	71209804	3	36	-2.85	mlv	3.08E-02
28.5	chr5	140963055	142590499	3	36	-2.85	mlv	1.78E-02
28.4	chr6	2072339	3965992	18	73	-1.61	mlv	2.25E-02
28.4	chr12	120458898	121004985	4	39	-2.62	mlv	2.24E-02
28.3	chr2	45643744	46649688	4	39	-2.62	mlv	3.30E-02
28.3	chr21	46705611	46846381	29	3	2.61	hiv	1.88E-03
28.2	chr4	116479033	119119770	29	3	2.61	hiv	1.70E-02
28.2	chr5	67838692	71626139	23	1	3.61	hiv	2.07E-02
28.2	chr15	89051854	89147874	23	1	3.61	hiv	1.10E-02
28.2	chr17	59881092	60062084	23	1	3.61	hiv	1.40E-02
28.2	chr4	175780626	182907412	44	10	1.71	hiv	1.70E-02
28.0	chr3	17757674	18271129	11	57	-1.89	mlv	3.26E-02
27.9	chr4	141093863	141256370	2	32	-3.18	mlv	1.80E-02
27.9	chr20	41814027	42731685	2	32	-3.18	mlv	4.73E-03
27.9	chr1	216590791	220202543	7	47	-2.19	mlv	6.41E-02
27.9	chr19	52971082	53443099	26	2	2.95	hiv	1.77E-02
27.9	chr6	124365828	125410702	26	2	2.95	hiv	2.86E-02
27.9	chr17	17125755	17636854	1	28	-3.83	mlv	1.50E-02
27.9	chr15	64694018	65308223	1	28	-3.83	mlv	1.20E-02
27.9	chr6	82526634	82745978	1	28	-3.83	mlv	2.73E-02
27.9	chr10	111818330	112618578	1	28	-3.83	mlv	1.61E-02
27.6	chr12	22733341	25117765	6	44	-2.29	mlv	3.24E-02
27.4	chr3	161038641	161386441	3	35	-2.82	mlv	4.18E-02
27.4	chr17	41012440	43045426	47	12	1.57	hiv	1.89E-02
27.4	chr3	197328368	197853794	8	49	-2.08	mlv	4.11E-02
27.2	chr4	91316463	95422991	33	5	2.17	hiv	2.43E-02
27.2	chr5	21041775	27684798	45	11	1.62	hiv	3.32E-02
26.9	chr10	64938686	69462483	28	3	2.57	hiv	2.56E-02
26.8	chrX	18890695	19812981	2	31	-3.15	mlv	1.95E-02
26.7	chr7	26240792	29571625	21	77	-1.49	mlv	6.37E-02
26.7	chr16	68993868	69115846	1	27	-3.79	mlv	8.19E-03
26.7	chr15	72806133	72950327	1	27	-3.79	mlv	1.94E-02
26.7	chr17	59694910	59876444	1	27	-3.79	mlv	2.36E-02
26.7	chr11	104603001	106919568	22	1	3.56	hiv	2.92E-02
26.7	chr12	759486	857169	22	1	3.56	hiv	4.80E-02
26.7	chr6	43264690	43688345	22	1	3.56	hiv	4.87E-02
26.7	chr15	73452971	73678628	22	1	3.56	hiv	1.60E-02
26.7	chr16	51805775	52519854	22	1	3.56	hiv	7.79E-03
26.7	chr15	57757865	58561240	1	27	-3.79	mlv	1.45E-02
26.7	chr1	54285997	55332577	1	27	-3.79	mlv	1.16E-01
26.5	chr9	133776457	134213839	25	2	2.91	hiv	3.14E-02
26.4	chr12	2237980	6362942	36	105	-1.23	mlv	5.53E-02
26.3	chr10	42454540	45541682	5	40	-2.39	mlv	3.33E-02
26.3	chr13	107714496	110639136	5	40	-2.39	mlv	1.44E-02
26.0	chr7	99455824	99855072	32	5	2.14	hiv	8.81E-02
26.0	chr14	41137726	44671440	32	5	2.14	hiv	4.12E-02
25.8	chr3	197935768	199205493	40	9	1.72	hiv	8.83E-02
25.7	chr13	76774517	76931603	2	30	-3.11	mlv	1.91E-02
25.6	chr9	79706818	81428417	6	42	-2.23	mlv	4.79E-02
25.5	chr1	196846926	196919054	1	26	-3.74	mlv	2.10E-01
25.5	chr8	62767300	62957500	1	26	-3.74	mlv	5.91E-02
25.5	chr18	44554916	44835039	1	26	-3.74	mlv	1.34E-02
25.5	chr22	15944311	16098818	1	26	-3.74	mlv	1.76E-02
25.5	chr11	32364767	32574206	1	26	-3.74	mlv	5.13E-02
25.4	chr4	95598928	102925146	69	27	1.08	hiv	5.76E-02
25.3	chr3	145478646	149292103	21	1	3.50	hiv	1.12E-01
25.3	chr6	42492739	42753758	21	1	3.50	hiv	9.16E-02
25.3	chr4	85844347	87592382	21	1	3.50	hiv	5.73E-02
25.3	chr9	101716957	103358624	21	1	3.50	hiv	5.47E-02
25.3	chr1	22677063	23532857	21	1	3.50	hiv	2.36E-01
25.2	chr11	95736520	99463626	24	2	2.86	hiv	6.00E-02
25.1	chr1	62731942	64648366	24	2	2.86	hiv	2.50E-01

25.1	chr6	45500211	47317585	9	49	-1.95	mlv	9.65E-02
24.7	chr6	44471005	45430023	31	5	2.10	hiv	1.09E-01
24.6	chr11	9593244	9738563	6	41	-2.21	mlv	7.57E-02
24.6	chr15	83093848	84051668	6	41	-2.21	mlv	3.91E-02
24.5	chr3	27393652	29696170	33	6	1.96	hiv	1.61E-01
24.5	chr17	37872308	38505378	46	13	1.45	hiv	7.35E-02
24.5	chr6	74518587	79837789	51	16	1.33	hiv	1.24E-01
24.4	chr17	45109333	45223766	1	25	-3.70	mlv	7.19E-02
24.3	chr6	83674510	86443292	26	3	2.48	hiv	1.28E-01
24.2	chr1	66569618	66678557	3	32	-2.72	mlv	3.99E-01
24.2	chr14	63873332	64998812	3	32	-2.72	mlv	1.01E-01
24.0	chr17	78073148	78596888	42	11	1.54	hiv	7.97E-02
23.9	chr8	6360591	11702713	25	81	-1.35	mlv	1.30E-01
23.5	chr4	160377031	164728336	30	5	2.06	hiv	1.41E-01
23.4	chr4	81264902	81335428	2	28	-3.03	mlv	1.39E-01
23.3	chr14	54683747	57762838	34	7	1.82	hiv	1.50E-01
23.2	chr4	88047607	90611444	13	56	-1.68	mlv	1.36E-01
23.2	chr3	161430736	169031485	56	20	1.19	hiv	3.19E-01
23.2	chr14	49373073	49653518	1	24	-3.65	mlv	1.56E-01
23.2	chr3	184159089	184461601	1	24	-3.65	mlv	3.08E-01
23.2	chr4	78954803	79807918	1	24	-3.65	mlv	1.35E-01
23.1	chr14	21604462	22409619	7	42	-2.06	mlv	1.56E-01
22.5	chr4	22915832	26361535	12	53	-1.71	mlv	1.69E-01
22.4	chr3	186015740	186447238	19	1	3.39	hiv	4.41E-01
22.3	chr16	64364178	65144503	2	27	-2.99	mlv	6.76E-02
22.2	chr3	195073119	196461256	7	41	-2.03	mlv	4.90E-01
21.8	chr3	126795730	130642226	31	89	-1.21	mlv	5.92E-01
