# Supplementary figures and images for: A novel scan statistics approach for clustering identification and comparison in binary genomic data
Source: BMC Bioinformatics. 2016 Sep 22;17(Suppl 11):320. doi: 10.1186/s12859-016-1173-8 (PMC5046198; doi:10.1186/s12859-016-1173-8)

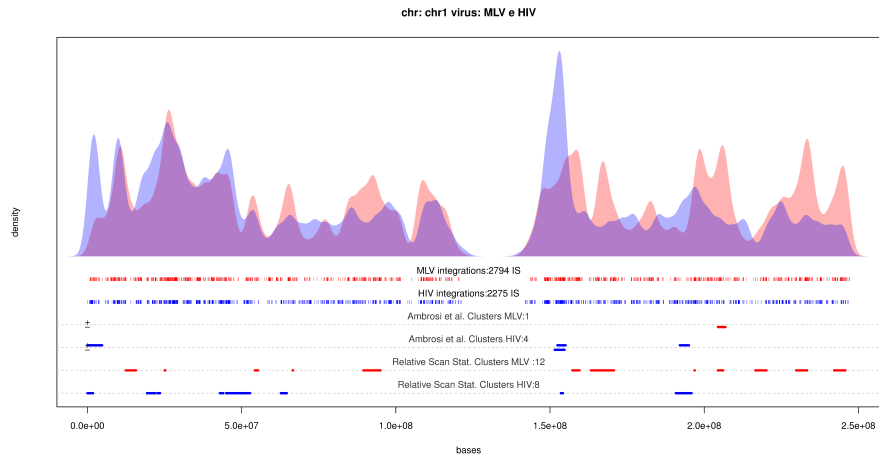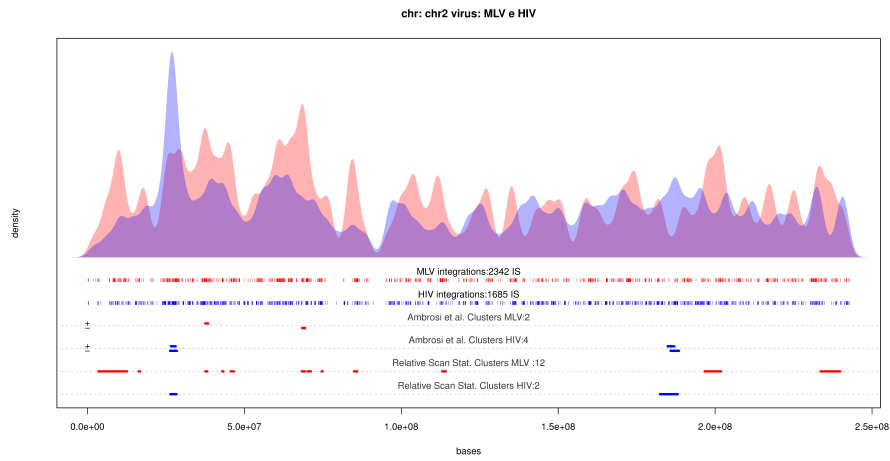

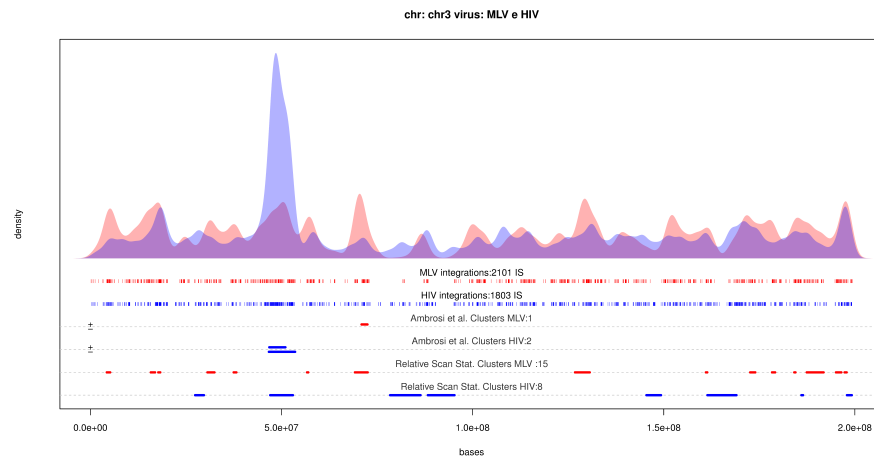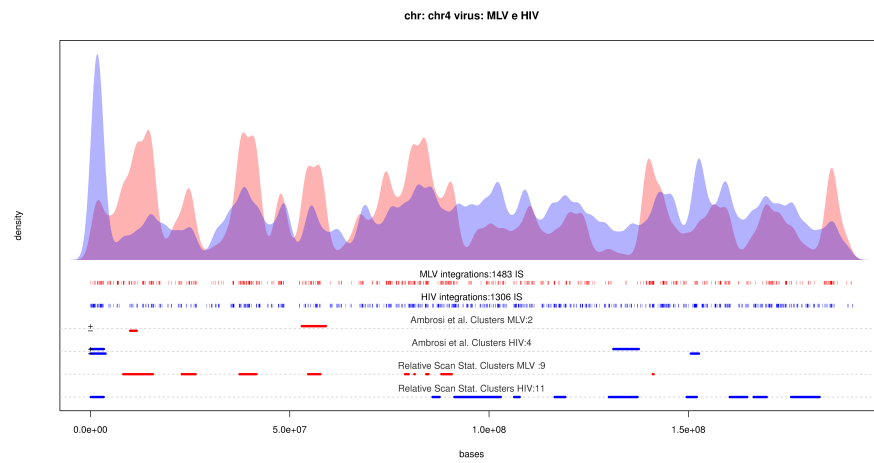

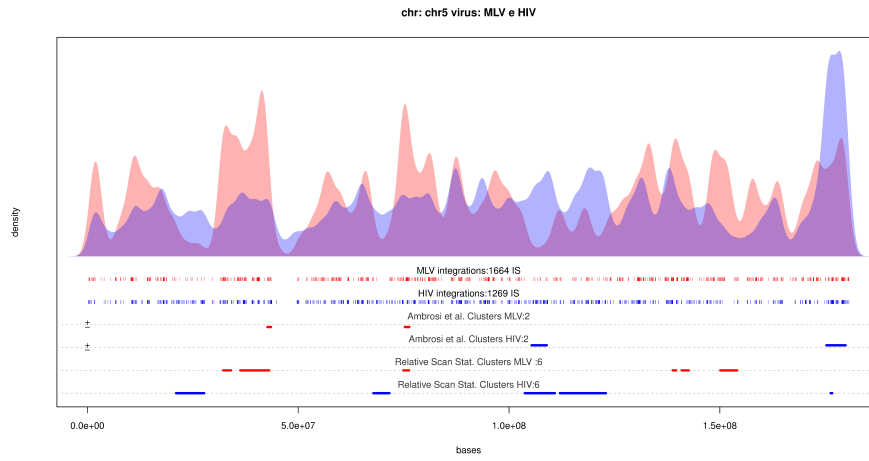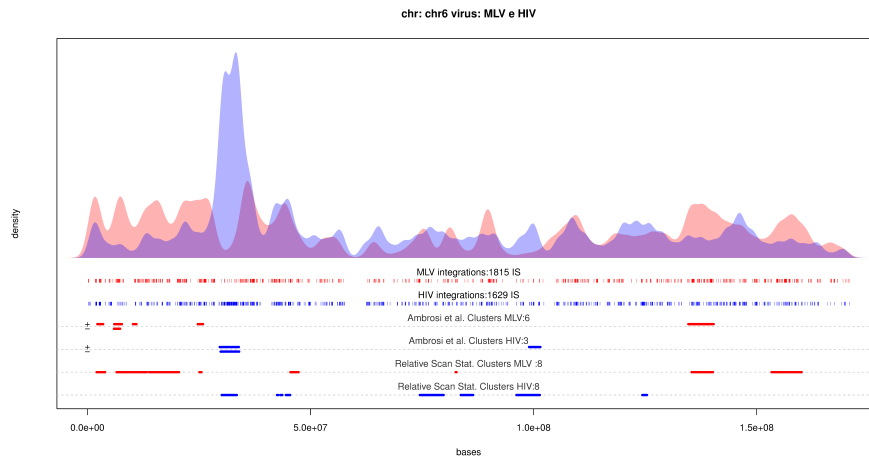

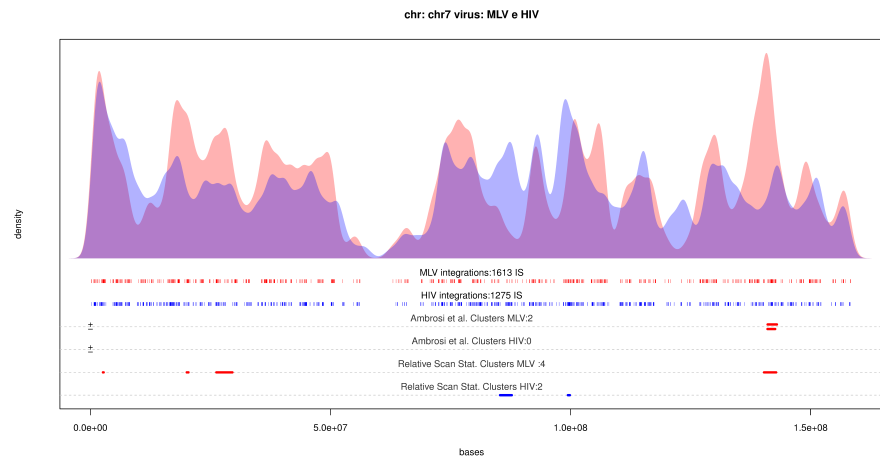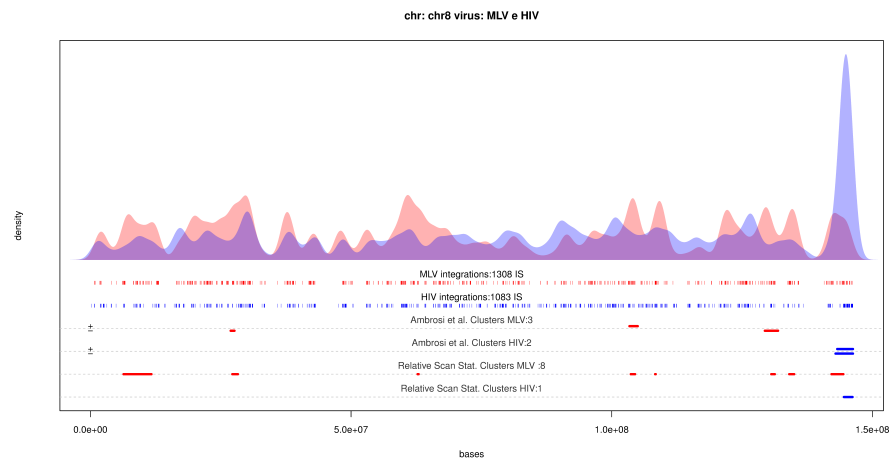

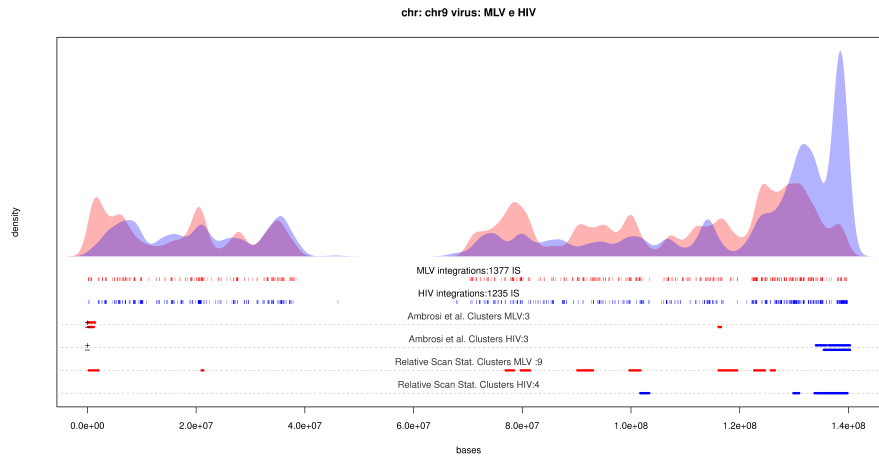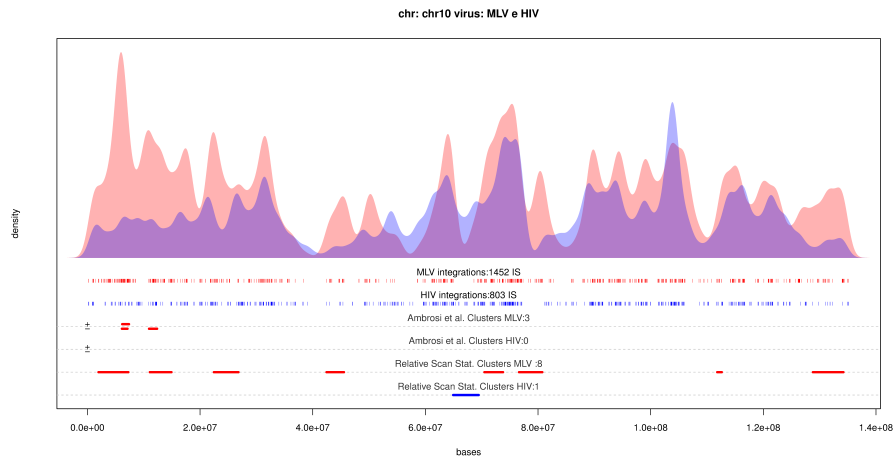

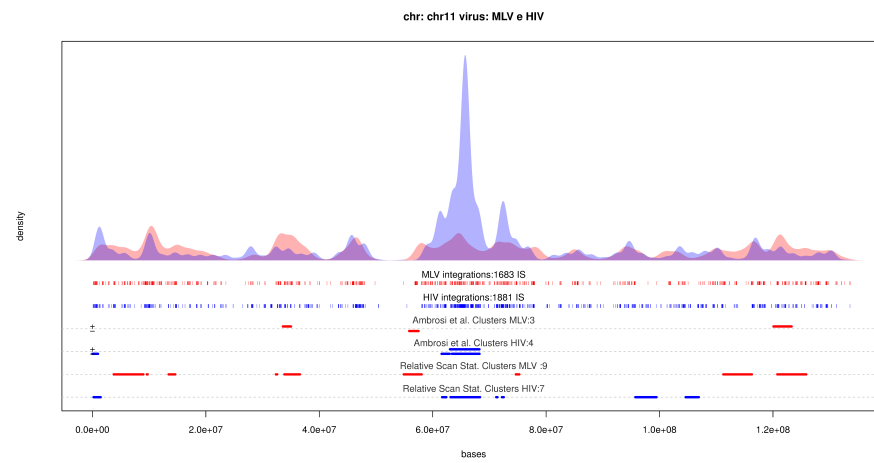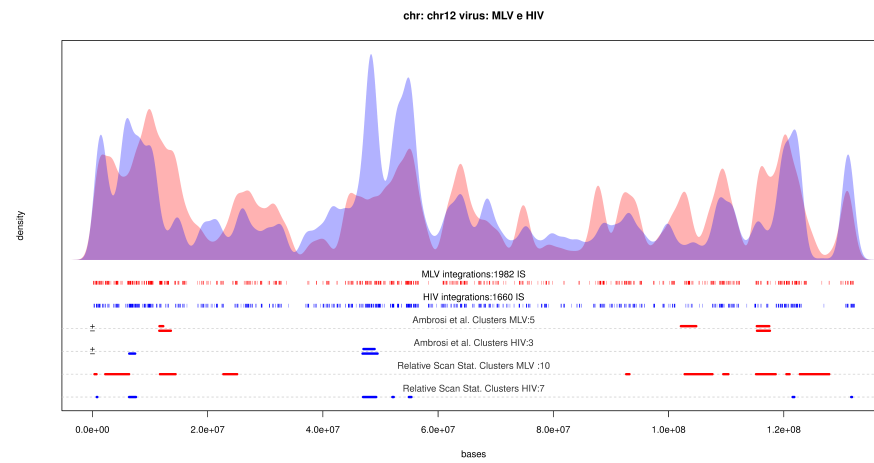

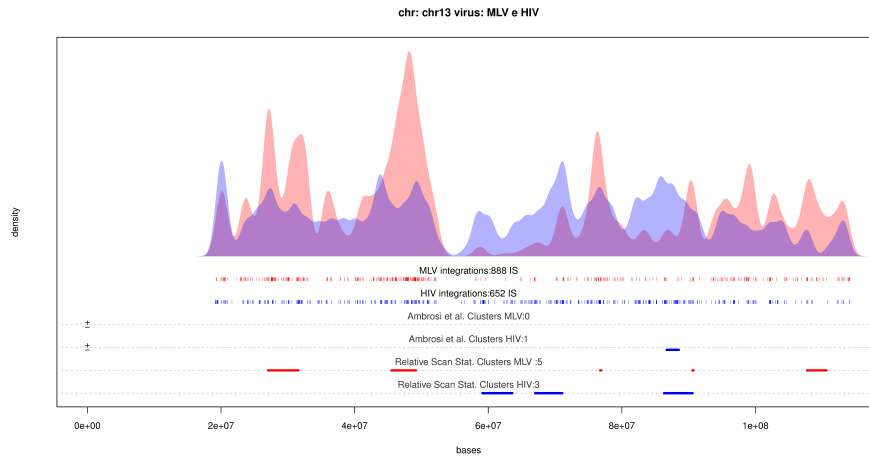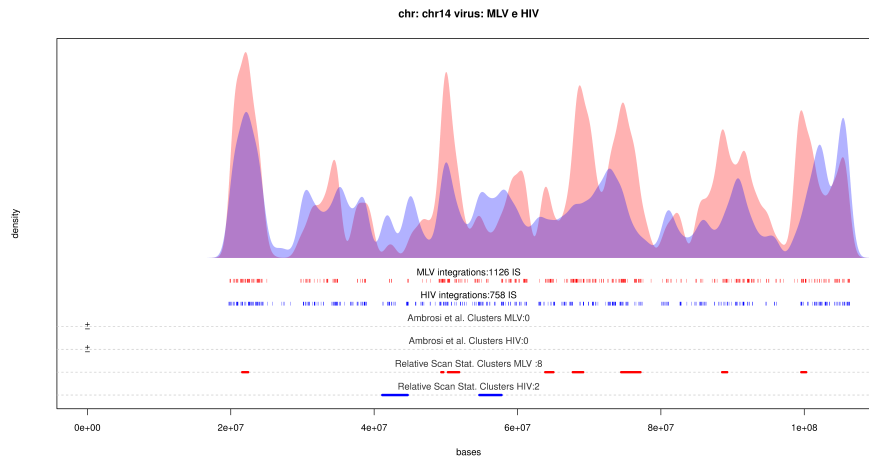

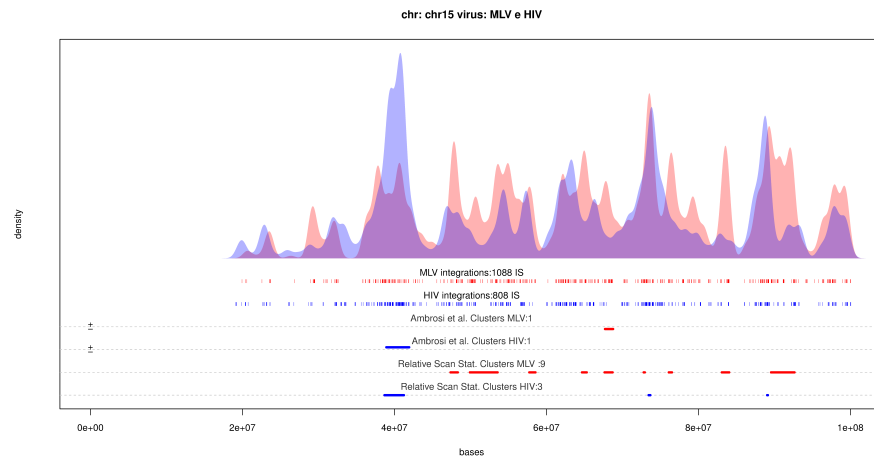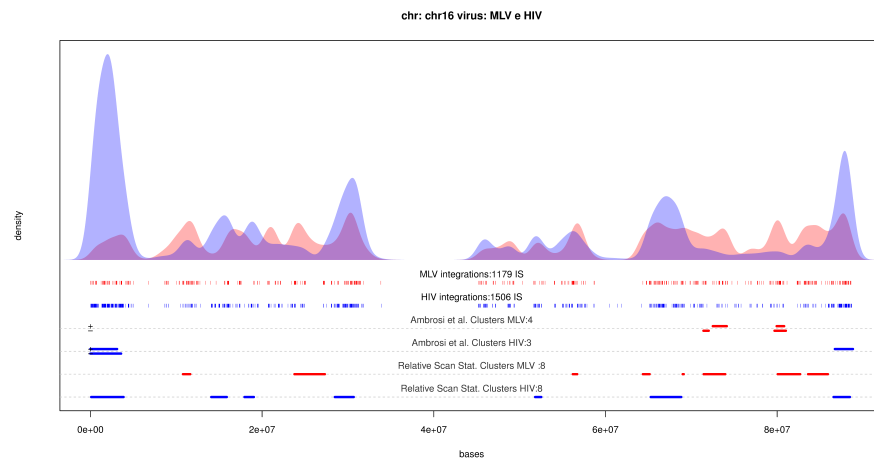

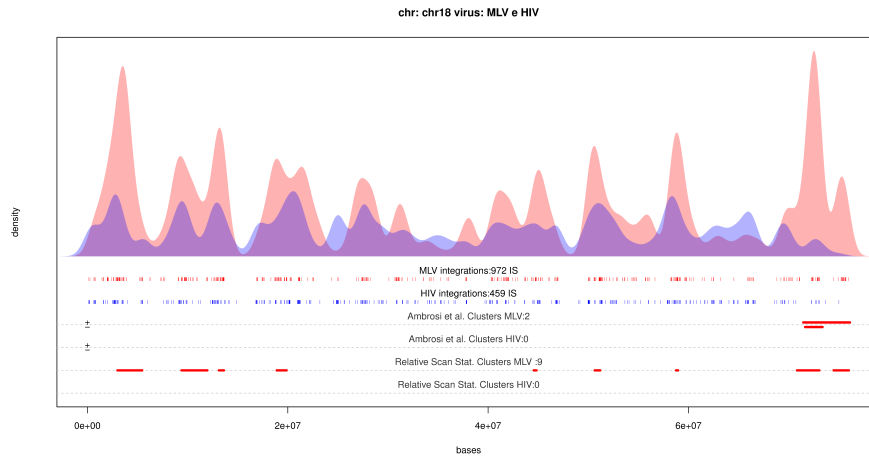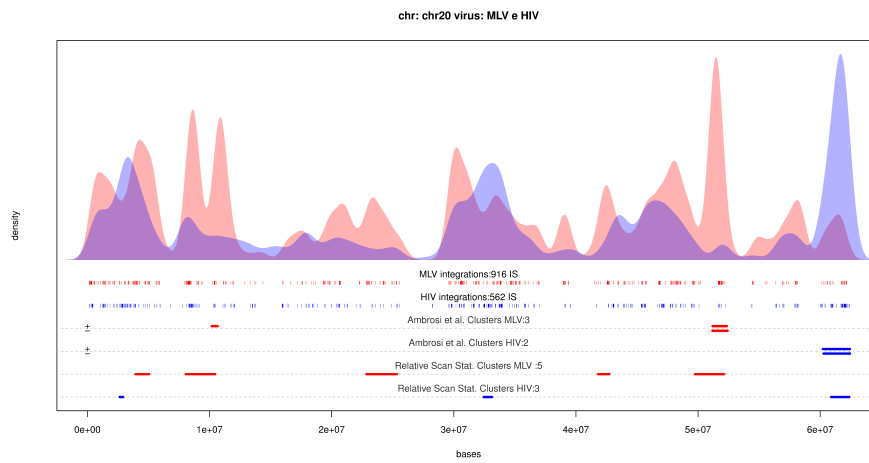

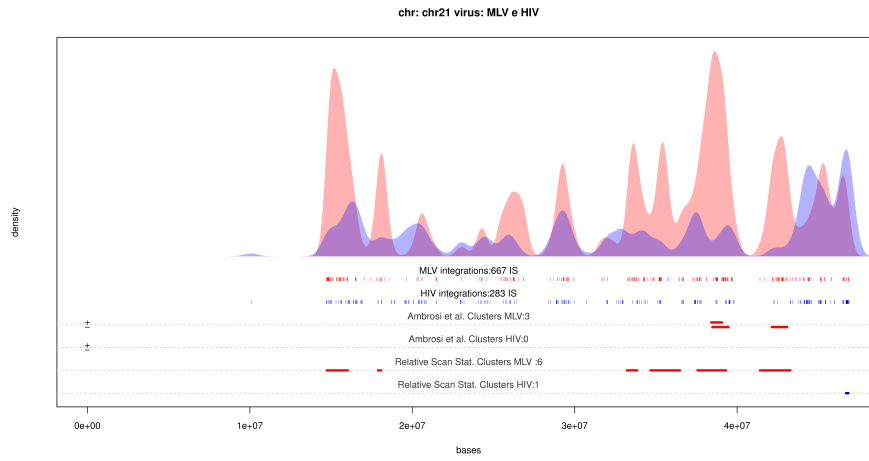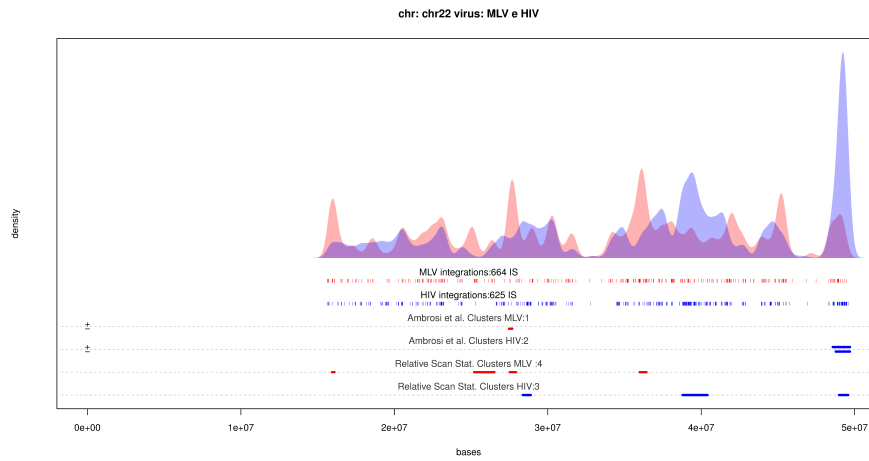

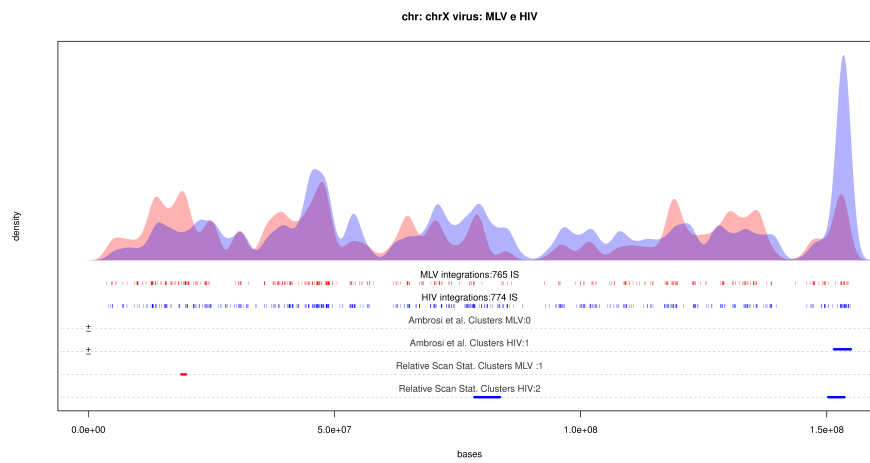

Supplement: Additional file 4 — Figure S1. Relative scan statistics results for remaining chromosomes. (PDF 5089 kb) [file 12859_2016_1173_MOESM4_ESM.pdf]
